# Endogenous erythropoietin signaling regulates migration and laminar positioning of upper-layer neurons in the developing neocortex

**DOI:** 10.1242/dev.190249

**Published:** 2020-10-07

**Authors:** Paul E. Constanthin, Alessandro Contestabile, Volodymyr Petrenko, Charles Quairiaux, Patrick Salmon, Petra S. Hüppi, Jozsef Z. Kiss

**Affiliations:** 1 Department of Fundamental Neurosciences, University Medical Center, University of Geneva, 1201 Geneva, Switzerland; 2 Division of Endocrinology, Diabetes, Hypertension and Nutrition, Department of Internal Medicine Specialties, University Hospital of Geneva, 1201 Geneva, Switzerland; 3 Department of Cell Physiology and Metabolism; Diabetes Center, Faculty of Medicine, University of Geneva; Institute of Genetics and Genomics in Geneva (iGE3), 1201 Geneva, Switzerland; 4 Department of Pediatrics, Faculty of Medicine, University Hospital of Geneva, 1201 Geneva, Switzerland

**Keywords:** Erythropoietin, Erythropoietin receptor, Neuronal migration, Cerebral cortex development

## Abstract

Erythropoietin (EPO), the hypoxia-inducible hematopoietic hormone, has well-established neuroprotective/neurotrophic roles in the developing central nervous system and the therapeutic potential of EPO has been widely explored in clinical studies for the treatment of perinatal hypoxic brain lesion, as well as prematurity. Here, we reveal that both EPO and Epo receptor (EPOR) are expressed in the developing rat somatosensory cortex during radial migration and laminar positioning of granular and supragranular neurons. Experimental deregulation of EPO signaling using genetic approaches results in aberrant migration, as well as permanent neuronal misplacement leading to abnormal network activity and protracted sensory behavioral deficits. We identify ERK as the downstream effector of the EPO signaling pathway for neuronal migration. These findings reveal a crucial role for endogenous EPO signaling in neuronal migration, and offer important insights for understanding how the temporary deregulation of EPO could result in migration defects that lead to abnormal behavior in the adult.

## Abstract (precis)

**Highlighted Article:** Experimental deregulation of erythropoietin signaling results in an aberrant migration and a permanent neuronal mispositioning, leading to abnormal formation of neuronal networks and sensory behavioral deficits later in life.

## INTRODUCTION

Radial migration of neocortical excitatory neurons from the ventricular/subventricular zone and their precise establishment in the six-layer neocortex is a highly-regulated multistep process (Kriegstein and Noctor, 2004; Rakic, 2007; Valiente and Marín, 2010). In the last decades, several genes and signaling pathways have been reported to play a crucial role during neuronal migration, correct final cell positioning and cortical lamination (see review by Valiente and Marín, 2010). Mutations, as well as deregulation of these molecular pathways, could result in cortical malformations with potentially severe neuropathological consequences, such as epilepsy, intellectual disability and autistic spectrum disorder (Bocchi et al., 2017; Sarkisian et al., 2008). The regulation and deregulation of migratory events are not well understood and represent an important challenge for future studies.

Erythropoietin (EPO), a 34 kDa glycoprotein, traditionally considered a hematopoietic cytokine, also has well-documented non-hematopoietic activity in various tissues including the central nervous system (see review by Alnaeeli et al., 2012). EPO and the EPO receptor (EPOR) have been detected in the rodent as well as in the human brain during early embryonic development (Juul et al., 1998a, 1999). Furthermore, the expression of both ligand and receptor appears to be dynamically regulated in both physiological and pathological conditions (Ogunshola and Bogdanova, 2013; Ott et al., 2015; Sanchez et al., 2009; Sirén and Ehrenreich, 2001). Of particular interest is that in animal models, EPO/EPOR are expressed in the neurogenic subventricular and ventricular zones of the cortex, in radial glia as well as in neurons attached to radial glial fibers (Knabe et al., 2004). Deletion of EPOR in a mouse model results in fewer progenitor cells and increased apoptosis (Tsai et al., 2006; Yu et al., 2002), suggesting that EPO signaling might play a role in the proliferation and survival of neuronal progenitors. Whether endogenous EPO signaling could be involved in the regulation of the radial migration of cortical neurons in the mammalian cerebral cortex remains largely unexplored.

The expression of EPO is hypoxia inducible, mainly through the hypoxia responsive element HIF-2 (Kapitsinou et al., 2010). In addition to hypoxia, tissue injuries were also shown to stimulate EPO expression (see review by Grasso et al., 2004) and EPO signaling has a well-established neuroprotective and neurotrophic role (see review by Ogunshola and Bogdanova, 2013). When interacting with its cognate transmembrane receptor (Epor), Epo induces dimerization and auto-phosphorylation of EPOR on its janus kinase 2 (JAK2) intracellular domain, leading to the activation of several downstream pathways including the transcription 5 (STAT5) pathway, the canonical kinase cascade Ras/Raf/MEK/ERK and the phosphoinositide 3-kinase (PI3K)/Akt pathway (Kretz et al., 2005; Lappin, 2003; Lombardero et al., 2011). Finally, in neuronal cells only, EPO signaling activates the nuclear factor IκB (Digicaylioglu and Lipton, 2001).

Given its neuroprotective effects (Brines and Cerami, 2005; Sirén and Ehrenreich, 2001), the therapeutic potential of EPO has been widely explored in recent years for the treatment of hypoxic brain lesions (Byts and Sirén, 2009; Gonzalez and Ferriero, 2009; Juul, 2000; Wu et al., 2016), as well as prematurity (Leuchter et al., 2014; Natalucci et al., 2016; Ohls, 2004), and several clinical studies have yielded promising results (He et al., 2008; Ohls, 2004; Zhu et al., 2009). However, the absence of comprehensive knowledge on the physiological roles of endogenous EPO signaling in brain development is a major drawback for developing efficient therapeutic strategies. In this study, we discovered that adequate levels of EPO signaling are required for the radial migration of cortical excitatory neurons. We focused on layer IV excitatory precursors in the rat somatosensory neocortex. We were able to identify a crucial role for this signaling pathway in regulating multipolar-to-bipolar transition, as well as radial glia-guided locomotion. The function of EPO in regulating cell migration is dependent on the Ras/Raf/MEK/ERK pathway. Importantly, we demonstrate that prenatal downregulation, as well as upregulation of intrinsic EPO signaling, will result in an aberrant migration and a permanent neuronal mispositioning, leading to an abnormal formation of neuronal networks and sensory behavioral deficits later in life.

## RESULTS

### Expression of EPO and its receptor EPOR in the developing neocortex

**Fig. 1.**
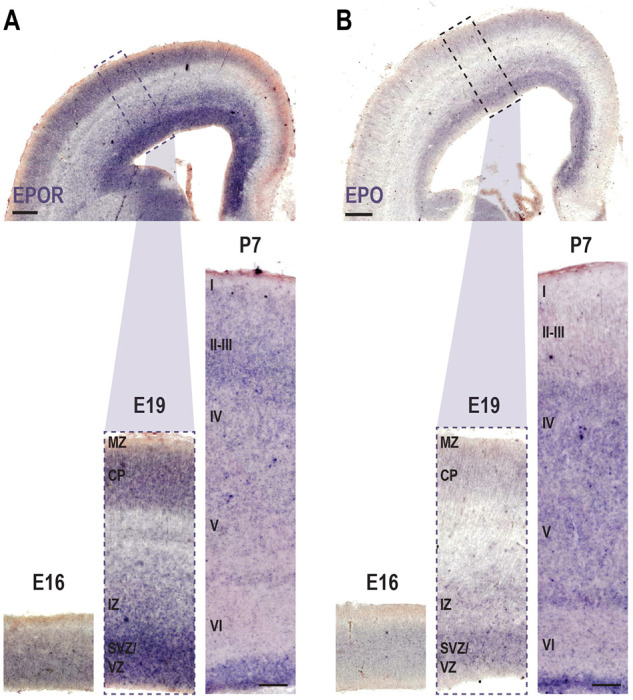
**EPO and EPOR expression is spatially and temporally regulated in the developing rat somatosensory cortex.** ISH for EPOR (A) and EPO (B) on coronal slices of the rat barrel cortex at E16, E19 and P7. Scale bars: 100 μm (A,B, lower panels) and 250 μm (A,B, upper panels).

To address the functions of EPO signaling in cortical cell migration, we first evaluated the expression of EPO and EPOR in the somatosensory cortex during radial migration and laminar positioning of granular and supragranular layer neurons. We performed *in situ* hybridization (ISH) for EPO and EPOR RNA expression in the rat somatosensory cortex at embryonic (E) day 16 and 19, as well as at postnatal (P) day 7 (Fig. 1A,B). E16 ISH revealed the presence of EPOR transcript throughout the developing cortex (Fig. 1A), with the exception of the marginal zone (MZ). Labeling for EPO was also detectable (Fig. 1B), though much less intense. The E19 neocortex exhibited a more differentiated pattern for EPOR transcript, with a strong labeling of the ventricular (VZ) and subventricular zones (SVZ), the lower part of the intermediate zone (IZ) and the cortical plate (CP). Immunostaining against EPOR (Fig. S1A) and ISH for EPO RNA at E19 (Fig. 1B) revealed a similar labeling pattern. In P7 neocortex, we found EPOR labeling in the area proximal to the ventricle, as well as in the upper layers of the cortex, whereas labeling for EPO transcript was the highest in the area proximal to the ventricle and in the deep layers of the cortex. Together, these results demonstrate the expression of both EPO and EPOR in the neocortex during late phases of radial migration, as well as during the early postnatal period.

### EPOR downregulation alters laminar positioning of upper-layer neurons

**Fig. 2.**
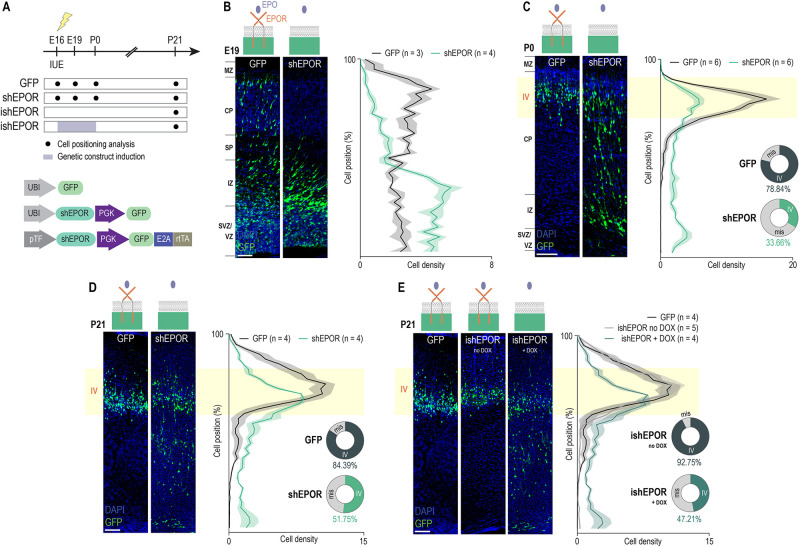
**EPOR downregulation results in neuronal misplacement.** (A) Experimental timeline. (B-D) Left panels: position of E16-electroporated cells in coronal slices from E19 (B), P0 (C) and P21 (D) with shEPOR plasmid. Right panels: quantification of the distribution of electroporated cells at E19 [GFP, *n*=3; shEPOR, *n*=4. Cell position×group interaction, *F*_(39, 195)_=15.2, *P*<0.0001; cell position main effect, *F*_(39, 195)_=12.28, *P*<0.0001; group main effect, *F*_(1, 5)_=0.011, *P*=0.9205], P0 [GFP, *n*=6; shEPOR, *n*=6. Cell position×group interaction, *F*_(39, 390)_=13.3, *P*<0.0001; cell position main effect, *F*_(39, 390)_=34.59, *P*<0.0001; group main effect, *F*_(1, 10)_=1.538, *P*=0.243] and P21 [GFP, *n*=4; shEPOR, *n*=4. Cell position×group interaction, *F*_(39, 234)_=7.4, *P*<0.0001; cell position main effect, *F*_(39, 234)_=27.58, *P*<0.0001; group main effect, *F*_(1, 6)_=0.2.154, *P*=0.1926]. In C and D, the proportion of neurons in layer IV are reported for GFP and shEPOR condition in pie charts. (E) Left panel: coronal slices from P21 E16-electroporated brains with GFP or ishEPOR (non-induced and induced between E16 and P0). Right panel: quantification of the distribution of electroporated cells along the cortex at P21 [control, *n*=4; ishEPOR no DOX, *n*=5; ishEPOR+DOX, *n*=4. *F*_(78, 390)_=3.966, *P*<0.0001; cell position main effect, *F*_(39, 390)_=41.07, *P*<0.0001; group main effect, *F*_(2, 10)_=1.055, *P*=0.3840]. The GFP condition shown in E is reported from D. Statistical significance was determined by two-way ANOVA followed by Bonferroni correction. Data are mean±s.e.m. *n*=number of analyzed brains (for each brain, more than 200 neurons were considered to define the mean). IUE, *in utero* electroporation. Scale bars: 20 μm (B); 50 μm (C); 100 μm (D,E).

To investigate whether EPO signaling affects radial migration, we constructed and screened small hairpin RNAs (shRNAs) coupled to a green fluorescent protein (GFP) and specifically targeting EPOR (shEPOR). We selected a construct that efficiently knocked down EPOR expression *in vitro* (Fig. S1B). We *in utero* electroporated the construct at E16 to downregulate EPOR in the neuronal precursors of layer IV of somatosensory cortex (Petrenko et al., 2015; Saito and Nakatsuji, 2001). Control electroporation was carried out with a GFP reporter plasmid (Fig. 2A) and with an off-target shRNA targeting an mRNA sequence unrelated to EPOR. Cell positioning analysis showed no statistically significant difference in the layer distribution of GFP and control shRNA-treated cells (Fig. S1C). ISH in unilateral shEPOR-electroporated brains showed a marked reduction of EPOR-RNA, especially in the IZ of the ipsi-electroporated hemisphere, in which electroporated precursors are located at E19 (Fig. S1D). Immunohistochemistry against EPOR in P21 brain slices obtained from E16 shEPOR-electroporated rats showed a decreased proportion of EPOR and GFP double-positive cells compared with controls (Fig. S1E-G). Post-hoc analysis of the laminar positioning of neurons at E19 showed a significant decrease in the proportion of shEPOR-electroporated cells reaching the CP (Fig. 2B). Although control electroporated cells were almost equally distributed throughout the migratory territory, extending from the SVZ to the upper portion of the CP, a large number of EPOR loss-of-function (LOF) cells accumulated in the IZ (Fig. 2B). The altered distribution of neurons was also observed at P0 (Fig. 2C), as well as at P21 (Fig. 2D), suggesting that at least for a subset of manipulated neurons the mispositioning was permanent (only 51.75% of shEPOR-electroporated neurons reached layer IV at P21) (Fig. 2D). This conclusion is further confirmed by data from cell positioning analysis at P35 that are comparable to that observed at P21 in Fig. 2 (Fig. S1H).

Immunostaining for the neuronal postmitotic marker SATB2 and the early differentiation marker TBR2 (also known as Eomes) performed on E19 brain slices of E16 GFP- or shEPOR-electroporated rats, revealed no difference between groups of double-positive cells (Fig. S2A-D). We observed no effect of EPOR downregulation at E17 and E19 on cell survival or cell cycle by quantification of cleaved caspase 3 and pKi67 staining of the electroporated cells compared with the control (Fig. S2E-J).

In order to test whether EPOR might also play a role in the neuronal migration of supragranular neurons, we electroporated E18 animals with either a GFP- or a shEPOR-overexpressing plasmid to target layer II/III pyramidal precursors. Cell positioning analysis at P3 showed a significant mispositioning of shEPOR-electroporated neurons (Fig. S3A,B).

We next tested whether a transient modulation of prenatal EPO signaling could lead to a permanent migration deficit. For this purpose, we used an inducible plasmid expressing an shRNA coupled to GFP and specifically targeting EPOR (ishEPOR) (Fig. 2A). Expression of this plasmid was controlled by a promoter under the influence of doxycycline (DOX) (see Giry-Laterrière et al., 2011 for more details about promotor efficiency and the timecourse of DOX activation) and validated *in vitro* (Fig. S4A). We electroporated ishEPOR at E16 and induced its expression between E16 and P0, sacrificed the animals at P21 and performed cell distribution analysis (Fig. 2A). As for the results obtained with the constitutive plasmid, we observed that ∼50% of ishEPOR^+^ neurons were correctly positioned in layer IV at P21, and a subset of labeled cells occupied a heterotopic position in infragranular layers (Fig. 2E). Thus, the transitory LOF of EPOR during radial migration could cause permanent mispositioning of neurons. To further confirm our results, we created a truncated form of EPOR by removing its intracellular domain and coupling it to a DOX-inducible promoter (iEPORT). E16 electroporation and induction between E16 and P0 of this dominant negative form of EPOR also resulted in a significant mispositioning of electroporated cells, whereas there was no effect on cell distribution in the absence of DOX (Fig. S4B,C). Taken together, these results demonstrate that an adequate level of EPOR expression is required for the laminar positioning of upper layer neurons.

### EPOR is required for the migration of upper-layer neurons in the intermediate zone as well as in the CP

**Fig. 3.**
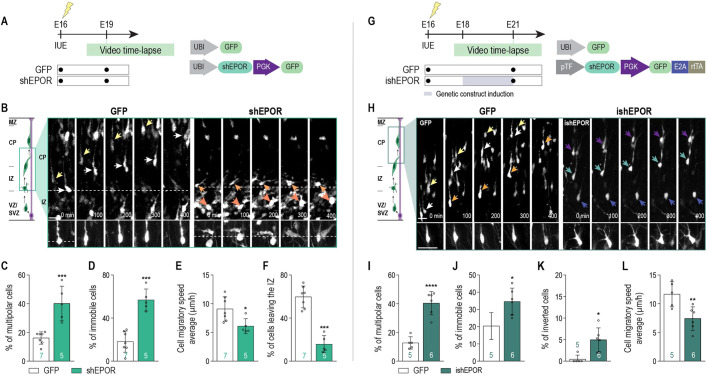
**EPOR downregulation alters neuronal migration in IZ and CP.** (A) Experimental timeline. (B) Single cell confocal time-lapse sequences of control and shEPOR-electroporated cells between the upper IZ and CP in freshly isolated slices. Examples of control neurons (left panel) exiting the IZ and shEPOR neurons (right panel) unable to exit the IZ are shown with arrows. (C) Proportion of cells that exhibited a multipolar morphology at one point during tracking time (control, *n*=7; shEPOR, *n*=5. *P*=0.0006). (D) Proportion of tracked cells that remained immobile during tracking time (control, *n*=7; shEPOR, *n*=5. *P*<0.001). (E) Mean neuronal migratory speed of tracked cells during tracking time (control, *n*=7; shEPOR: *n*=5. *P*=0.0194). (F) Proportion of cells leaving the IZ during tracking time (control, *n*=7; shEPOR, *n*=5. *P*<0.001). (G) Experimental timeline. (H) Time-lapse sequences from freshly isolated slices of control and i shEPOR-electroporated cells in the CP. Examples of control neurons (left panel) migrating at a normal speed and ishEPOR neurons (right panel) unable to migrate correctly are shown with arrows. (I) Proportion of cells that exhibited a multipolar morphology at one point during tracking time (control, *n*=5; ishEPOR, *n*=6. *P*<0.0001). (J) Proportion of tracked cells that remained immobile during tracking time (control, *n*=5; ishEPOR, *n*=6. *P*=0.0145). (K) Proportion of cells that exhibited an inverted migratory direction at one point during tracking time (control, *n*=5; ishEPOR, *n*=6. *P*=0.0077). (L) Mean neuronal migratory speed of tracked cells during tracking time (control, *n*=5; ishEPOR, *n*=6. *P*=0.009). Data are mean±s.d. Statistical significance was determined using an unpaired two-tailed Student's *t*-test. *P<0.05, **P<0.01, ***P<0.001, ****P<0.0001. *n*=number of analyzed brains (for each brain, more than 50 neurons were considered to define the mean), and these values are featured at the base of the bars in the graphs in C-F and J-M. IUE, *in utero* electroporation. Scale bars: 10 μm.

In order to study the role of EPOR on cell migration, we performed real-time confocal time-lapse imaging. As we observed that many EPOR LOF cells accumulated in the intermediate zone (IZ)/upper SVZ, we focused on this region at E19 on E16 shEPOR- or GFP-electroporated brains (Fig. 3A). Analysis of the migrating behavior of shEPOR-electroporated cells in the upper part of the IZ revealed significantly fewer cells transitioning from their multipolar state to the bipolar one compared with GFP-electroporated cells (Fig. 3B,C). Furthermore, significantly more shEPOR-electroporated cells remained immobile during the entire duration of the recording (Fig. 3B,D). Finally, we observed significantly lower migratory speeds in the shEPOR-electroporated cells compared with controls (Fig. 3E). This ultimately resulted in fewer cells exiting the IZ towards the CP (Fig. 3B,F).

In order to evaluate the role of EPOR in the gliophilic locomotion of neurons along the radial glia in the CP, we performed real-time confocal time-lapse imaging at E21. ishEPOR-overexpressing plasmid was induced at E18 to minimize the LOF effect on the multipolar-to-bipolar transition (Fig. 3G). Single cell time-lapse imaging revealed that significantly fewer ishEPOR-electroporated cells remained immobile or presented a multipolar phenotype (Fig. 3H-J). We also observed that a slightly higher proportion of ishEPOR-electroporated cells presented an inversion of the leading process from the basal (i.e. towards pial surface) to apical (i.e. towards ventricle) direction and moving back towards the IZ (Fig. 3K). LOF cells took pauses more frequently during locomotion, leading to a significantly lower average migratory cell speed in the CP (Fig. 3L). As the attachment of migrating neurons to radial glia is crucial for leading process stability (Elias et al., 2007), we evaluated the adherence of migrating neurons to radial glia. We measured the Euclidian distance between the leading process and the nearest radial glia fibers revealed by immunostaining against nestin. The quantitative analysis revealed that the average distance between ishEPOR-electroporated cells and radial glia fibers significantly increased compared with controls, particularly in the portion of the leading process more proximal to the cell body (Fig. S5A,B).

In order to explore whether the functional EPO/EPOR binding is necessary for these functions, we co-electroporated two plasmids overexpressing shEPOR or EPO (Fig. S7A; see Fig. S7C for *in vitro* validation of the EPO-overexpressing plasmid). Such co-electroporation results in the co-expression of the two plasmids in more than 90% of neuronal precursors (Fig. S6A-C; Bocchi et al., 2017). We observed that co-electroporated cells did not migrate properly and that a significant percentage of them could not reach the CP (Fig. S7B). These results suggest that even if neuronal EPO production is increased, the EPOR signaling pathway is not properly activated when EPOR is downregulated and the neuronal migration remains altered. We conclude that functional binding between EPO and its receptor EPOR is required during neuronal migration. Taken together, these results indicate that EPOR expression in migrating cells is required for proper migration speed, as well as for maintaining leading process stability.

### Modulation of EPO ligand in migrating neurons impairs gliophilic locomotion

**Fig. 4.**
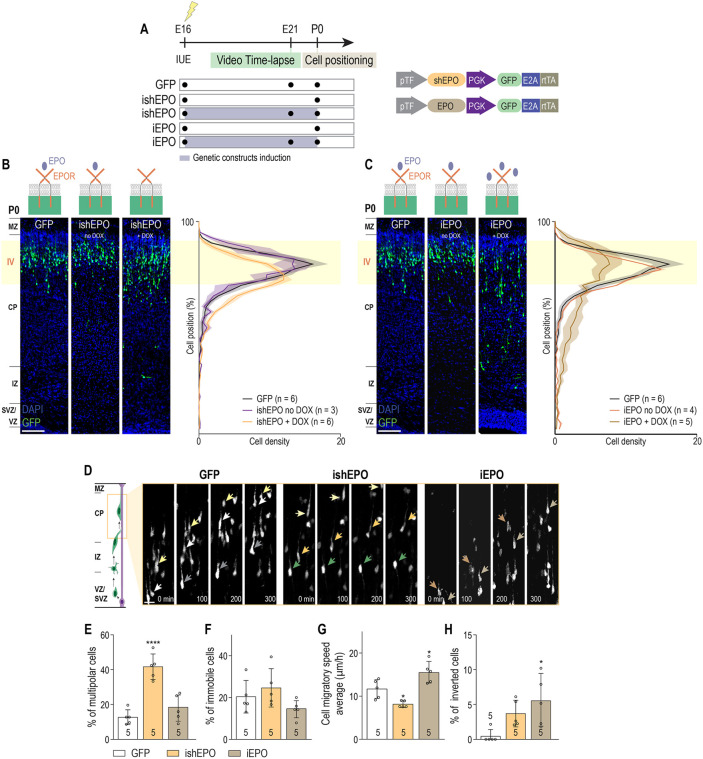
**Cell-autonomous EPO signaling is necessary for correct gliophilic locomotion in the CP.** (A) Experimental time-line. (B) Left panel: coronal slices from P0 E16-electroporated brains with GFP and ishEPO (with and without DOX). Right panel: quantification of the distribution of electroporated cells along the cortex at P0 [GFP, *n*=6; ishEPO no DOX, *n*=3; ishEPO+DOX, *n*=6. Cell position×group interaction, *F*_(78, 468)_=3.317, *P*<0.0001; cell position main effect, *F*_(39, 468)_=73.5, *P*<0.0001; group main effect, *F*_(2, 12)_=0.7201, *P*=0.5066]. (C) Left panel: coronal slices from P0 E16-electroporated brains with GFP and iEPO (with and without DOX). Right panel: quantification of the distribution of electroporated cells along the cortex at P0 [GFP, *n*=6; iEPO no DOX, *n*=4; iEPO+DOX, *n*=5. Cell position×group interaction, *F*_(78, 468)_=3.623, *P*<0.0001; cell position main effect, *F*_(39, 468)_=56.88, *P*<0.0001; group main effect, *F*_(2, 12)_=0.0, *P*>0.9999]. (D) Time-lapse sequences from freshly isolated slices of control, iEPO and ishEPO-electroporated cells in the CP. Examples of control neurons (left panel) migrating at a normal speed (arrows) and ishEPO and iEPO neurons (middle and right panels) unable to migrate correctly (arrows) are shown. (E) Proportion of cells that remained multipolar during tracking time [control, *n*=5; ishEPO, *n*=5; iEPO, *n*=5. *F*_(2, 12)_=27.43, *P*<0.0001]. (F) Proportion of tracked cells that remained immobile during tracking time [control, *n*=5; ishEPOR, *n*=5; iEPO, *n*=5. *F*_(2, 12)_=2.375, *P*=0.1352]. (G) Mean neuronal migratory speed of tracked cells during tracking time [control, *n*=5; ishEPO, *n*=5; iEPO, *n*=5. *F*_(2, 12)_=17.56, *P*=0.003]. (H) Proportion of cells that inverted their migratory direction during tracking time [control, *n*=5; ishEPO, *n*=5; iEPO: *n*=5. *F*_(2, 12)_=5.503, *P*=0.0201]. The GFP conditions shown in B, C and D are reported from Fig. 2C or Fig. 3H, respectively. Statistical significance was determined by two-way (B,C) or one-way (E-H) ANOVA followed by Bonferroni multiple comparisons test. Data are mean±s.d. *P<0.05, ****P<0.0001. *n*=number of analyzed brains [for each brain more than 50 neurons (E-H) or more than 200 neurons (B,C) were considered to define the mean], and for E-H these values are featured at the base of the bars in the graphs. IUE, *in utero* electroporation. Scale bars: 100 μm (B,C) and 10 μm (D).

As we observed the expression of both the EPO ligand and EPOR during radial migration, and functional EPO/EPOR binding is needed, we tested the effects of EPO LOF, as well as gain-of-function (GOF) in migrating neurons. We electroporated late neuronal progenitors of the future layer IV with either an inducible plasmid overexpressing an shRNA specifically targeting EPO (ishEPO) or an inducible plasmid overexpressing EPO ligand (iEPO) (Fig. 4A). Expression of both plasmids was controlled by a promoter under the influence of DOX. The efficiency of these constructs was validated *in vitro* [Fig. S7C (iEPO) and Fig. S7E (ishEPO)], as well as *in vivo* by ISH on P0 brain slices (Fig. S7D). Post-hoc analysis of cell positioning at P0 showed significantly fewer cells reaching the future layer IV after an electroporation with ishEPO or iEPO when DOX was given in drinking water at E16 (Fig. 4B,C).

Single cell imaging of neuronal migratory behavior at E19 revealed no differences between controls and ishEPO or iEPO during multipolar-to-bipolar transition phase (Fig. S7F-H). On the other hand, during the locomotion phase in the CP at E21, we found a significantly increased percentage of multipolar/immobile neurons and a decreased mean cell speed in ishEPO-electroporated brains compared with controls (Fig. 4D-G). These results were comparable to that obtained with the induction of ishEPOR-plasmid at E18 (Fig. 3H-L). The quantitative analysis of the migratory behavior of EPO-overexpressing cells in the CP revealed an increased cell speed compared with controls (Fig. 4G). Moreover, a significantly higher proportion of iEPO^+^ cells presented an inverted migratory direction compared with controls (Fig. 4H).

These results demonstrate that both downregulation and upregulation of EPO ligand in migrating layer IV neurons perturbs radial glia guided locomotion but not multipolar-to-bipolar transition in the upper IZ. Moreover, they raise the possibility that EPO, at least partially, cell-autonomously regulates locomotion.

### EPO signaling regulates the migration of neurons via ERK activation

**Fig. 5.**
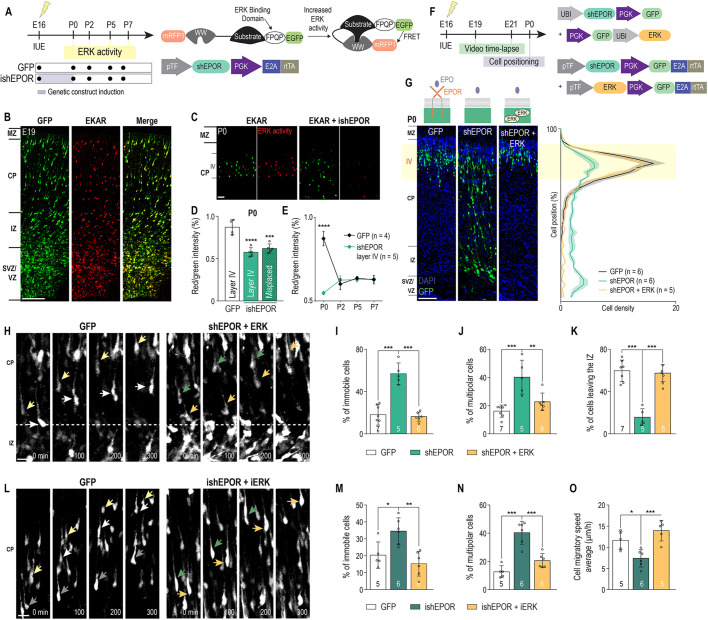
**ERK upregulation rescues shEPOR-induced migratory deficit.** (A) Experimental timeline for EKAR-based experiments. (B) Coronal slice from an E19 control brain electroporated with an activity reporter to monitor ERK activity (EKAR), showing that ERK is active in migrating neurons. (C) Coronal slices from P0 E16-electroporated brains with EKAR (left panel) and ishEPOR+EKAR (right panel). (D) Quantification of ERK activity (intensity ratio RFP/GFP) in layer IV control and layer IV and misplaced ishEPOR conditions at P0 [control, *n*=4; ishEPOR, *n*=5. *F*_(2, 11)_=54.24, *P*<0.0001]. (E) Quantification of ERK activity (intensity ratio RFP/GFP) in layer IV control (black) and layer IV ishEPOR (green) conditions at P0 (control, *n*=4; ishEPOR, *n*=5), P2 (control, *n*=3; ishEPOR, *n*=5), P5 (control, *n*=4; ishEPOR, *n*=4) and P7 (control, *n*=3; ishEPOR, *n*=5). *F*_(3, 25)_=25.03, *P*<0.0001; time main effect, *F*_(3, 25)_=6.781, *P*=0.0017; group main effect, *F*_(1, 25)_=19.77, *P*=0.0002. (F) Experimental timeline for ERK-based rescue experiments. (G) Left: coronal slices from P0 brains electroporated at E16 with GFP, shEPOR or shEPOR+ERK. Right: quantification of the proportion of cells at each level of the cortex at P0 [control, *n*=6; shEPOR, *n*=6; shEPOR+ERK, *n*=5. Cell position×group interaction, *F*_(78, 546)_=8.845, *P*<0.0001; cell position main effect, *F*_(39, 546)_=71.36, *P*<0.0001; group main effect, *F*_(2, 14)_=2.211, *P*=0.1465]. (H) Time-lapse sequences from E19 freshly isolated slices of control and shEPOR+ERK-electroporated cells between the upper IZ and CP. Examples of control neurons (left panel) and shEPOR+ERK-electroporated cells migrating correctly are indicated (arrows). (I) Proportion of tracked cells that remained immobile during tracking time [control, *n*=7; shEPOR, *n*=5; shEPOR+ERK, *n*=6. *F*_(2, 15)_=39.06, *P*<0.0001]. (J) Proportion of cells that remained multipolar during tracking time [control, *n*=7; shEPOR, *n*=5; shEPOR+ERK: *n*=6. *F*_(2, 15)_=14.66, *P*=0.0003]. (K) Proportion of cells exiting the IZ during tracking time [control, *n*=7; shEPOR, *n*=5; shEPOR+ERK, *n*=6. *F*_(2, 15)_=42.1, *P*<0.0001]. (L) Time-lapse sequences from E21 freshly isolated slices of control and shEPOR+ERK-electroporated cells in the CP. Examples of control neurons (left panel) and shEPOR+ERK-electroporated neurons (right panel) migrating at a normal speed are indicated (arrows). (M) Proportion of tracked cells that remained immobile during tracking time [control, *n*=5; ishEPOR, *n*=6; shEPOR+ERK, *n*=6. *F*_(2, 14)_=10.27, *P*=0.0018]. (N) Proportion of cells that remained multipolar during tracking time [control, *n*=5; ishEPOR, *n*=6; shEPOR+ERK, *n*=6. *F*_(2, 14)_=30.37, *P*<0.0001]. (O) Mean neuronal migratory speed of tracked cells during tracking time [control, *n*=5; ishEPOR, *n*=6; shEPOR+ERK, *n*=6. *F*_(2, 13)_=12.4, *P*=0.0010]. The GFP conditions shown in G, H and L are reported from Fig. 2C, Fig. 3G and Fig. 3K, respectively. Statistical significance was determined by one-way (D,I-K,M-O) and two-way (E) ANOVA followed by Bonferroni correction. Data are mean±s.d. *P<0.05, **P<0.01, ***P<0.001, ****P<0.0001. *n*=number of analyzed brains [for each brain, more than 50 neurons (I-K,M-O) or more than 200 neurons (D,E,G) were considered to define the mean] and in I-K and M-O, these values are featured at the base of the bars in the graphs. IUE, *in utero* electroporation. Scale bars: 20 μm (B); 10 μm (C,H,L); 50 μm (G).

In order to identify potential downstream molecular mechanisms, we first evaluated the presence of ERK activity during neuronal migration. We used a FRET-based sensor of ERK activity (EKAR) expressing EGFP and red fluorescent protein (RFP) (Harvey et al., 2008). Upon its attachment to the phosphorylated form of ERK, EKAR increases the expression of RFP, thus increasing the cellular RFP/EGFP ratio (Fig. 5A). We electroporated EKAR at E16 and then quantified the intensity ratio of RFP/GFP. First, ERK-activity was observed in electroporated cells at E19 (Fig. 5B). Moreover, co-electroporation of EKAR with a GFP-deprived version of ishEPOR showed a significant decrease of ERK activity in the electroporated neurons at P0 (Fig. 5C,D). After the removal of DOX from drinking water at P0, the ERK activity returned to the same level as the control group (Fig. 5E). Thus, ERK activity in migrating cells is regulated, at least in part, by EPOR receptor activation.

We then attempted to rescue the migratory phenotype resulting from EPOR downregulation by co-electroporating shEPOR at E16 with a construct expressing a constitutively active form of ERK (Fig. 5F). ERK overexpression completely rescued the migratory phenotype at P0 (Fig. 5G). On the other hand, overexpression of the Akt pathway through a constitutively active plasmid failed to rescue the migratory misplacement at P0, and actually worsened the phenotype (Fig. S8A,B). E16 electroporation of constitutively active ERK alone induced a significant overmigration of neuronal progenitors, leading to a misplacement of these cells at P0 in the future layers II-III instead of layer IV (Fig. S8C,D). In contrast, electroporation of constitutively active Akt showed a significant misplacement of neuronal progenitors with an arrest of migration in the upper IZ (Fig. S8C,D). Real-time confocal time-lapse imaging at E19 on E16 ERK and shEPOR co-electroporated cells, revealed a normalization of the proportion of immobile cells in the IZ compared with the control (Fig. 5H,I). Furthermore, multipolar-to-bipolar transition in the upper IZ was also rescued, leading to a normal exit of electroporated cells from the IZ (Fig. 5J,K). Comparable observations were made in the CP at E21, in which cells co-electroporated with shEPOR and ERK presented a rescued migratory behavior, and a recovery of locomotion along radial glia (Fig. 5L-O). Post-hoc analysis at E21 on rescued cells revealed a phenotype that was no different from control cells with regards to the distance between the leading process and radial glia in the CP (Fig. S5A,B). These observations give strong support to the hypothesis that ERK is a downstream effector of EPO signaling in regulating radial migration and neuronal differentiation.

### Prenatal downregulation of EPO-signaling during radial migration leads to an altered differentiation and a reduced neuronal activation of layer IV neuronal precursors in early adulthood

Positioning of neurons during development is an important definer of neuronal identity and function. To explore the consequences of transient perturbations in EPO signaling on neuronal differentiation, we analyzed post-hoc E16 ishEPOR-electroporated brains in which our construct was induced only prenatally (Fig. S9A). Neuronal differentiation was tested at P21 by immunostaining for NeuN, a neuronal differentiation marker, for Cux1, an upper layer marker, and for CTIP2 (also known as Bcl11b), a lower layer marker (Fig. S9A). We quantified immunostaining for these three markers in misplaced cells, as well as in those that reached layer IV. We found that a significant proportion of ishEPOR^+^ neurons, whether they reached layer IV or were misplaced, did not display either NeuN or Cux1 staining (Fig. S9B-E). Moreover, misplaced ishEPOR-electroporated neurons were not CTIP2^+^, indicating that their position in lower layers might not affect their phenotype (Fig. S9F,G). We also performed dendritic arborization reconstruction of electroporated cells using Neurolucida software (MBF Bioscience, v11.02.1), separating cells that reached layer IV and misplaced cells. ishEPOR-electroporated cells presented a significant increase of first order dendrites, as well as an increased number of dendritic nodes per cell, and were longer but thinner than controls (Fig. S9H-J). Moreover, dendritic arborization of misplaced cells closely resembled that of layer IV cells, pointing to a direct effect of the prenatal EPO-signaling downregulation on dendritogenesis rather than an indirect role because of the migratory misplacement. We then measured the density of protrusions present in the second order dendrites of electroporated cells at P21 when synaptic contacts have started to be established between cells. ishEPOR-electroporated cells in layer IV exhibited significantly fewer protrusions, and there were even fewer in misplaced cells (Fig. S9K,L). Together, these results suggest that transient downregulation of EPO signaling affects later steps of neuronal differentiation, including dendritic development and the formation of excitatory synapses.

**Fig. 6.**
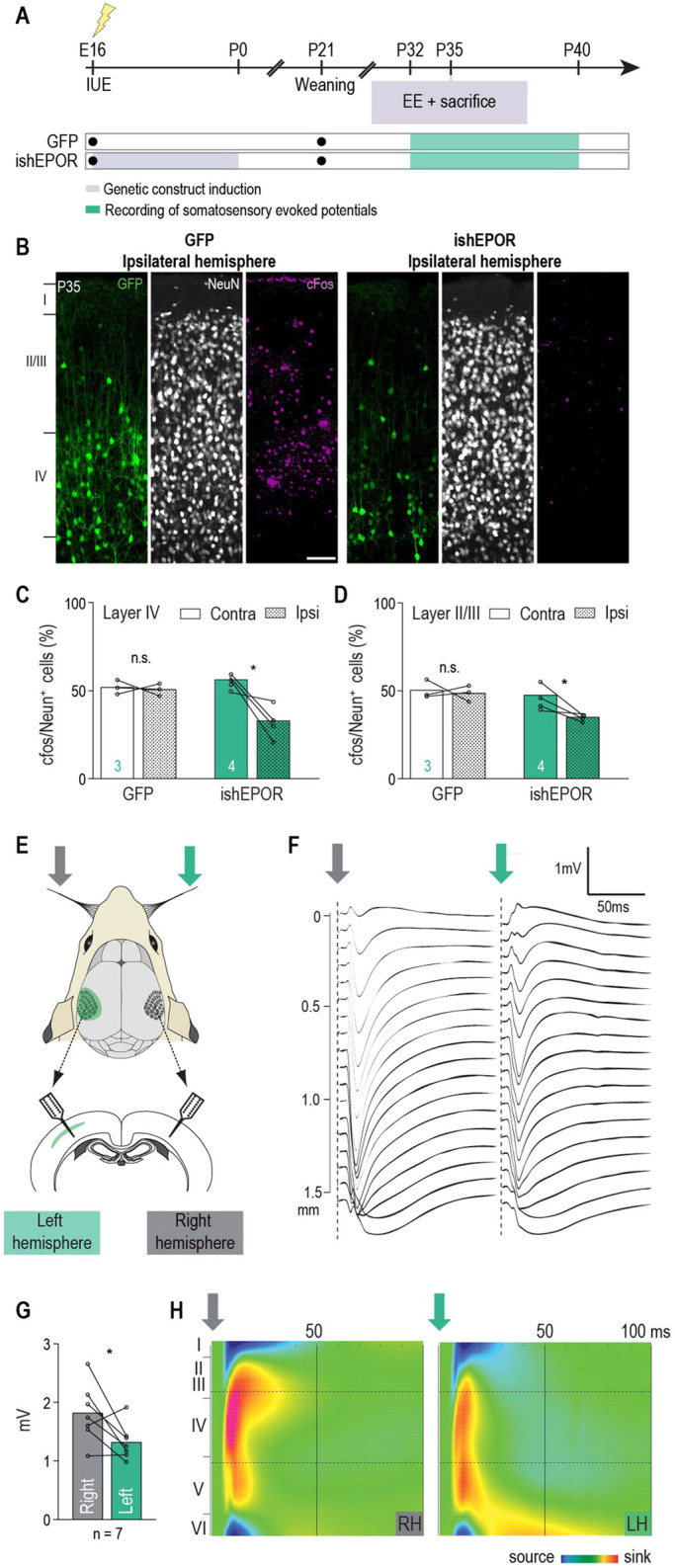
**Prenatal perturbation of intrinsic EPO signaling leads to impaired cortical activity later in life.** (A) Experimental timeline. (B) cFos-staining at P35 of electroporated sides for GFP (left panel) and ishEPOR (right panel) conditions after placement in an enriched environment (EE). (C) Percentage of cFos^+^ NeuN cells in layer IV in control and ishEPOR conditions after placement in an EE at P35 (control, *n*=3 brains; ishEPOR, *n*=4 brains. *P*=0.6885 for GFP and *P*=0.0436 for ishEPOR). (D) Percentage of cFos^+^ NeuN cells in layer II-III in control and ishEPOR conditions after placement in an EE at P35 (control, *n*=3 brains; ishEPOR, *n*=4 brains. *P*=0.9665 for GFP and *P*=0.0389 for ishEPOR). (E) Schematic of intracortical recordings of contralateral whisker-evoked LFPs in the ishEPOR hemisphere and in the opposite hemisphere. (F) Average LFP traces in the right (grey arrow) and left (green arrow, ishEPOR side) hemispheres in response to 50 contralateral whisker stimulations in an illustrative animal. Arrow positions indicate the onset of contralateral whisker stimulations. (G) Average maximal LFP amplitudes measured from the maximal negative to maximal positive peak across the depth of the cortex in the left (ishEPOR, green) and right (non-injected, grey) hemispheres (*n*=7 animals. *P*=0.043). (H) Current-source density plots calculated from the illustrative LFPs recordings shown in E. Although reduced in amplitude, current source and sink positions are not dramatically altered. In C,D, *n* values are features at the base of the bars in the graphs. The dotted bars in C and D indicate the ipsilateral condtion. Statistical significance was determined by paired two-tailed Student's *t*-tests. *P<0.05, n.s., not significant. IUE, *in utero* electroporation. Scale bar: 100 μm.

We next tested the hypothesis that prenatal perturbation of EPO signaling in migrating neurons might have an impact on circuit development in the affected hemisphere. To evaluate the activity level of neurons, E16-electroporated P35 animals were placed in an enriched environment for 80 min and then immediately sacrificed (Fig. 6A). We used immunostaining for c-Fos as a neuronal activity reporter and determined the percentage of NeuN^+^ cells that also contained c-Fos immunoreactivity in layer IV of the barrel cortex as described previously (Bocchi et al., 2017). The proportion of c-Fos^+^ cells of the contralateral (not electroporated) hemisphere was used as the control. As expected, there was no difference in the proportion of c-Fos^+^ neurons between the two hemispheres in GFP-electroporated animals. In contrast, ishEPOR-electroporated hemispheres showed significantly fewer cFos^+^ cells in their layer IV compared with their wild-type counterpart (Fig. 6B,C). The activity-attenuating effect of ishEPOR on cortical neurons observed in the electroporated layer IV expands to the adjacent layers II and III (Fig. 6D). No significant difference in c-Fos positivity was found between the control sides of both groups. To further explore cortical circuit functions, we recorded somatosensory evoked potentials (SEP) across all layers of the left and right S1 cortices in response to 50 contralateral whisker stimulations in anesthetized P32 to P40 rats (Fig. 6E-H). The results from this supported our cFos findings as they revealed a 27% smaller amplitude of the evoked local field potentials (LFPs) amplitudes in the ishEPOR-electroporated hemisphere compared with the opposite hemisphere (Fig. 6G). These results demonstrate that prenatal perturbation of intrinsic EPO-signaling during radial migration leads to altered neuronal activation in the affected hemisphere later in life. Prenatal downregulation of EPOR in migrating neurons leads to long-term alterations in sensory functions that could be rescued with ERK overexpression.

**Fig. 7.**
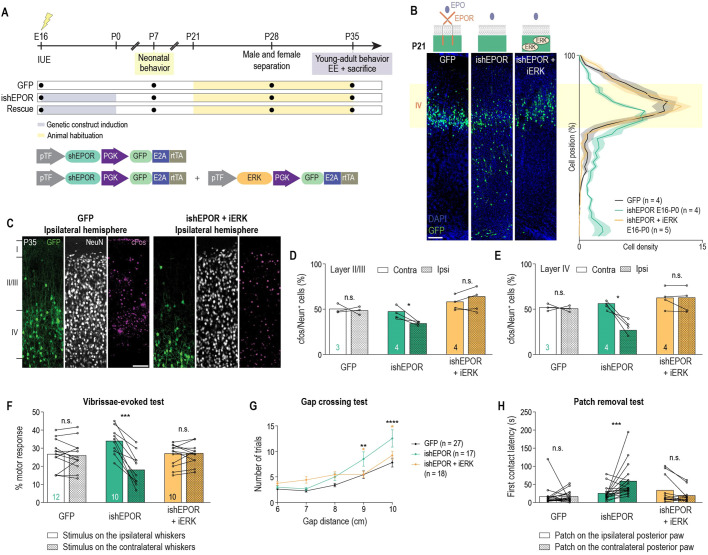
**Prenatal ERK upregulation rescues prenatal ishEPOR-induced perturbed neuronal activity and sensory functions.** (A) Experimental timeline. (B) Left panel: coronal slices from P21 E16-electroporated brains with GFP, ishEPOR or ishEPOR+iERK. Right panel: quantification of the distribution of electroporated cells along the cortex at P21 [GFP, *n*=4; ishEPOR, *n*=4; ishEPOR+iERK, *n*=5. Cell position×group interaction, *F*_(78, 390)_=4.508, *P*<0.0001; cell position main effect, *F*_(39, 390)_=46.45, *P*<0.0001; group main effect, *F*_(2, 10)_=2.485, *P*=0.1330]. (C) cFos-staining at P35 of electroporated sides for GFP (left panel) and iEPORsh+iERK (right panel) conditions after placement in an EE. (D) Percentage of cFos^+^ NeuN cells in layer II-III in control, ishEPOR and iEPORsh+iERK conditions after placement in an EE at P35 (GFP, *n*=3 brains; ishEPOR, *n*=4; ishEPOR+iERK, *n*=4. *P*=0.9665 for GFP, *P*=0.0389 for ishEPOR, and *P*=0.3215 for iEPORsh+iERK). (E) Percentage of cFos^+^ NeuN cells in layer IV in control, ishEPOR and iEPORsh+iERK conditions after placement in an EE at P35 (GFP, *n*=3 brains; ishEPOR, *n*=4; ishEPOR+iERK, *n*=4. *P*=0.6885 for GFP, *P*=0.0436 for ishEPOR, and *P*=0.4214 for iEPORsh+iERK). (F) Percentage of motor response during a vibrissae-evoked behavior test at P7 for both sides for control, ishEPOR and iEPORsh+iERK conditions (GFP, 12 animals; ishEPOR, ten animals; iEPORsh+iERK, ten animals. *P*=0.5961 for GFP; *P*<0.0001 for ishEPOR, and *P*=0.9066 for iEPORsh+iERK). (G) Number of trials to cross the gap with respect to the distance of the gap for GFP, ishEPOR and ishEPOR+ERK groups at P35 [GFP, 27 animals; ishEPOR, 17 animals; iEPORsh+iERK, 18 animals. Gap×group interaction, *F*_(8, 193)_=2.612, *P*=0.0098; gap main effect, *F*_(4, 193)_=35.95, *P*<0.0001; group main effect, *F*_(2, 193)_=10.63, *P*<0.0001]. (H) First contact latency in patch-removal task at P35 for both sides for control, ishEPOR and iEPORsh+iERK conditions (GFP, 27 animals; ishEPOR, 17 animals; iEPORsh+iERK, 18 animals. *P*=0.9940 for GFP, *P*=0.0139 for ishEPOR, and *P*=0.636 for iEPORsh+iERK). The GFP and ishEPOR conditions shown in B are reported from Fig. 2D and E. The GFP condition shown in C is reported from Fig. 6B. Statistical significance was determined by two-way ANOVA (B,G) or paired two-tailed Student's *t*-tests (D-F,H). Data are mean±s.e.m. *n*=number of analyzed brains [for each brain, more than 200 neurons (B) were considered to define the mean] or number of mice (D-H), and these values are featured at the base of the bars in the graphs in D-F. The dotted bars in D and E indicate the ipsilateral condtion. IUE, *in utero* electroporation. n.s., not significant. Scale bars: 100 μm.

In order to confirm that EPO signaling-related migration errors might play a role in altered cortical development, we examined whether rescuing migration errors with ERK overexpression could also rescue the observed altered differentiation and reduced neuronal activation. A DOX-inducible ERK-overexpressing plasmid (iERK) was generated and co-electroporated at E16 with ishEPOR, and DOX was given from E16 to P0 (Fig. 7A). Cell position analysis at P21 showed that there was no difference with GFP-electroporated brains, which demonstrated that the prenatal rescue with ERK is long lasting (Fig. 7B). Cellular differentiation and maturation, tested by NeuN- (Fig. S9A-C) and Cux1-immunostaining (Fig. S9A,D,E), were also rescued. Furthermore, the cellular morphology (Fig. S9H-J) and the density of dendritic protrusions (Fig. S9K,L) of cells co-electroporated with ishEPOR and iERK were not different from control cells. Neuronal activity at P35, tested by c-Fos analysis, revealed that there was no difference between both hemispheres in layer IV and layers II and III, as was observed in the controls (Fig. 7C-E).

As there is evidence that a migration deficit of a small fraction of neurons in the dorsomedial cortex has been sufficient to perturb animal behavior (Bocchi et al., 2017), we next tested whether prenatal downregulation of the EPO signaling in the somatosensory cortex could also generate long-term sensory deficits or related behavioral alterations in adulthood. Animals were electroporated at E16 with ishEPOR and received DOX from E16 to P0. We first used a modified version of the unilateral vibrissae-evoked test for neonatal pups (Sullivan et al., 2003) to explore whisker sensitivity at P7 (Fig. 7F). In this test, no differences were observed in the proportion of motor response to stimulation between the ipsilateral and contralateral whiskers in control electroporated animals. In contrast, ishEPOR-electroporated pups displayed a reduced proportion of motor response to the stimulation on the contralateral whiskers (connected to the electroporated hemisphere) compared with the ipsilateral whiskers (Fig. 7F). To test whether this behavioral deficit persisted until adulthood, animals were tested at P35 with the gap-crossing test and the patch-removal task, which are two known tests of the activity of the somatosensory cortex. ishEPOR-electroporated animals exhibited an increase in the number of trials to cross the gap in the gap-crossing test, becoming significant for a gap of 9 cm (Fig. 7G). In the patch-removal task, ishEPOR-electroporated animals presented an increase in the first contact latency of the contralateral paw compared with the ipsilateral one; no such difference was found in the control group (Fig. 7H). We conclude that a transient perturbation of EPO signaling and the subsequent perturbation of the migration and connectivity of a subpopulation of layer IV neurons is sufficient to produce long-term somatosensory deficit.

We finally tested whether the rescue of the migratory and network phenotypes (with ERK) resulted in a recovery of the behavior of the animals. A unilateral vibrissae-evoked test at P7 revealed no asymmetry between sides in the motor response to stimuli (Fig. 7F). Later on, a gap-crossing test at P35 showed no differences between ishEPOR/iERK and control animals in the number of trials needed to cross the gap (Fig. 7G). Last, there was no asymmetry observed in the first contact latency (Fig. 7H). These results give support to the hypothesis that perturbed EPO signaling-induced migrations errors are crucial for altered circuit formation and behavior.

### Prenatal overexpression of EPO ligand also affected long-term neuronal positioning, activation and sensory functions

We also tested the long-lasting effects of a transient prenatal EPO overexpression with our iEPO plasmid (Fig. S10A). P21 post-hoc analysis revealed permanent mispositioning of a significant percentage of iEPO^+^ cells (Fig. S10B), P35 c-Fos analysis revealed an increased proportion of c-Fos^+^ neurons in layers III and IV of the ipsilateral hemisphere in iEPO-electroporated mice (Fig. S10C,D). Finally, iEPO-electroporated mice exhibited a sensorial deficit in the vibrissae-evoked test at P7 (Fig. S10E) and in the gap-crossing test but not in the patch-removal test at P35 (Fig. S10F,G). These results reveal that not only downregulation but also upregulation of EPO signaling could lead to altered neocortical development and functional deficits.

## DISCUSSION

Our results reveal that EPO signaling in the brain is required for proper radial migration and neocortical layer formation. We provide the first evidence that transient prenatal perturbations of EPO or EPOR expression in migrating neuronal precursors of cortical upper layer excitatory neurons can result in the aberrant positioning or permanent misplacement of cortical neurons, thus causing cortical networks to perform abnormally. This is paralleled by altered somatosensory functions and behavior in young animals that persist into adulthood. These findings reveal a role for intrinsic EPO signaling during cortical layer formation and provide a new framework for understanding how deregulated intrinsic EPO signaling in pathological conditions, including perinatal hypoxic/ischemic lesions or encephalopathy of prematurity, might contribute to migration deficits and altered circuit formation in the cerebral cortex.

Consistent with previous reports in human (Juul et al., 1998b, 1999) and different animal models (Chen et al., 2007; Yu et al., 2002), we found that both EPO and EPOR were expressed in the neurogenic VZ of the developing cortex. Furthermore, we demonstrated that transcripts for both components were present in other layers, including the SVZ, IZ, CP and supragranular and infragranular layers during radial migration, and during the early postnatal period. The expression of EPOR was required for at least two different stages of the radial migration of upper layer excitatory neurons, as demonstrated by our knockdown experiments using shEPOR and ishEPOR. First, we found that downregulating EPOR expression in migrating cells resulted in the altered polarization and accumulation of multipolar cells in the IZ. The multipolar-to-bipolar transition is a crucial checkpoint for radial migration, as it determines the number of cells that can exit and move up to the CP (Boitard et al., 2015; Kriegstein and Noctor, 2004; Rakic, 2007; Valiente and Marín, 2010). Indeed, our real-time imaging provided direct evidence for the reduced exit of EPOR LOF cells from the SVZ, similar to that we observed previously after upregulating canonical Wnt signaling in these cells (Boitard et al., 2015). Thus, EPO signaling appears to contribute to the complex regulatory network of multitudes of signaling molecules that controls radial migration via multipolar-to-bipolar transition (Valiente and Marín, 2010). Second, we showed that EPOR was also essential for gliophilic locomotion of neurons. We found that decreasing EPOR levels in migrating layer IV neurons resulted in reduced migratory speed and delayed arrival of neurons in the CP. Importantly, both downregulation and upregulation of EPO ligand in migrating neurons perturbed radial glia-guided locomotion. We found that the downregulation of EPO reduced migratory speed, whereas its overexpression led to an increased migratory speed of neurons. These results give strong support to the hypothesis that EPO signaling might contribute to the regulation of this phase of radial migration either cell-autonomously or through a paracrine action.

In contrast to gliophilic migration through the CP, perturbation of EPO expression did not affect the multipolar-to-bipolar transition in the upper SVZ and IZ. The reason for this difference between the two phases of radial migration is not clear. One possible explanation is that within the multipolar-to-bipolar transition zone, in a high cell density context, sufficient and saturating concentrations of EPO ligand may be provided by non-electroporated cells via paracrine secretion. Thus, upregulation or downregulation of EPO ligand in a subpopulation of migrating cells would be compensated. This might not be the case during radial glia-guided locomotion, when the cell-autonomous mode of EPO signaling might be dominant in individual neuronal precursors and cannot be compensated by paracrine release of EPO from more distant cells when EPO ligand production is perturbed. Thus, similar to the canonical Wnt signaling (Bocchi et al., 2017), adequate levels of EPO signaling during locomotion are required for the proper pace of radial migration. Interestingly, and contrary to the transient alteration of Wnt signaling (Bocchi et al., 2017) causing migration delays, the reversible downregulation of intrinsic EPO signaling also led to a permanent misplacement of neuronal progenitors, similar to the ones observed in some pathological conditions, such as periventricular heterotopia, that have been linked with epilepsy as well as mental retardation later in life (Sarkisian et al., 2008). These data highlight the importance of intrinsic neuronal EPO signaling during crucial periods of the radial migration of upper layer neurons, and warrant further research investigating a potential role for this signaling in pathological conditions, both as a cause and as a potential treatment.

The Ras/Raf/MEK/ERK and the PI3K/Akt pathways are well-known downstream effectors of EPOR activation that have been implicated in regulating cell migration (Aguilar et al., 2014; Lester et al., 2005; Liu et al., 2013; Park et al., 2014). Our single cell imaging revealed that ERK activity was high during radial migration, including during the multipolar-to-bipolar transition as well as the gliophilic locomotion in the CP. We also showed that in these contexts, EPO signaling acts upstream of the ERK pathway. Most importantly, overexpression of ERK prevented the migratory phenotype that we observed with EPOR and EPO LOF neurons. Remarkably, ERK activity also significantly decreases after birth in control neurons as reported previously (Chen et al., 2006; Juul et al., 1998b, 1999; Knabe et al., 2004; Liu et al., 1997). This might suggest that the role of the Ras/Raf/MEK/ERK pathway in neuronal migration might be limited to prenatal cortical development. Our results suggest that EPO signaling might be one of the main regulators of ERK signaling in upper layer neurons during their migration. This hypothesis warrants further research to investigate the interconnection between EPO signaling and the Ras/Raf/MEK/ERK pathway. On the other hand, upregulation of the PI3K/Akt pathway was unable to rescue our migratory deficit. Hence, the regulatory effects of EPO signaling on cell migration are based, at least partially, on the activation of ERK pathway.

In contrast to previous studies (Tsai et al., 2006; Yu et al., 2002), we did not observe any decrease in the survival and proliferation in our model of EPOR LOF. It is important to emphasize that although previous experiments were conducted in genetically modified animals presenting a complete knockout of EPOR in the developing brain, we introduced shRNA plasmids in only a subpopulation of neuronal precursors. In addition, the LOF of EPOR in this study was considerably less severe, as the production of EPOR is only partially downregulated. Therefore, neurons might have retained enough EPOR production to sustain their own survival and proliferation. Another, plausible explanation is that the effects of shEPOR occur after the phase of proliferation when the impact of EPO signaling on cell survival is decreased. We cannot exclude the possibility that the altered migratory phenotype is due, at least partially, to an abnormal neuronal differentiation of neuronal precursors, although this is unlikely. We observed that downregulation of prenatal EPOR production did not affect the expression of the neuronal postmitotic marker SATB2 and the early differentiation marker TBR2. On the other hand, a significant number of EPOR LOF cells did not express the final neuronal marker NeuN, or the upper-layer-specific marker Cux1, when EPOR was prenatally downregulated. This might suggest a role for EPO signaling in the late-phase final differentiation of neuronal progenitors, paralleling its role in the maturation of late-stage erythrocyte progenitors. Noteworthy in this respect, practically all cells with downregulated EPOR exhibited altered late-phase differentiation patterns but only a subpopulation was mispositioned. Thus, perturbed differentiation does not necessary lead to abnormal migration. It is also possible that misplaced shEPOR^+^ cells in lower layers could differentiate as lower layer neurons and express layer-specific markers. However, this is unlikely as misplaced neurons lacked CTIP2 staining, suggesting that the position of misplaced neurons in lower layers does not affect the neuronal phenotype.

We found that neurons with transient manipulations of EPOR display reduced neuronal activity in adults. Furthermore, we observed a reduced overall neuronal activation in the affected hemispheres, suggesting that EPOR downregulation leads to somatosensory dysfunctions. Whether the abnormal behavior stems from altered activity of the misplaced neurons or the overall decrease in the activity of the barrel cortex due to a lack of excitatory pyramidal neurons remains to be determined. These phenotypes can all be rescued by normalizing migration with the upregulation of the downstream signaling element ERK. These findings give strong support to the hypothesis that altered integration of even a subset of neurons in the developing cortex due to modified EPO signaling during pregnancy is sufficient to cause persisting behavioral abnormalities.

Therapy with EPO has shown promising results against cerebral ischemic lesions in both animal and human studies. However, the notion that EPO treatment has a positive effect on the neurodevelopment of prematurely born infants remains controversial (Bierer et al., 2006; Check Hayden, 2016; Natalucci et al., 2016) and prospective studies are still ongoing (Check Hayden, 2016; Rangarajan and Juul, 2014). Our study shows an important effect of intrinsic EPO signaling during neuronal migration. It also reveals that not only downregulation but also upregulation of EPO signaling might be detrimental for long-term development and could lead to the emergence of migration-related neuropsychiatric disorders. This suggests that both the dose, as emphasized by Natalucci et al. (2016), and the timing of EPO treatment is of crucial importance and warrant further research, notably on a possible effect of postnatal intrinsic EPO signaling in the brain.

## MATERIALS AND METHODS

### Animals

The experimental procedures described here were conducted in accordance with Swiss laws and were previously approved by the Geneva Cantonal Veterinary Authority. Female and male Wistar rats were housed in the institutional animal facility under standard 12 h light/12 h dark cycles with food and water *ad libitum*. All rats were weaned at P21 and separated by gender at P28. Where indicated, DOX (Sigma-Aldrich) was administered (1 mg/ml) in drinking water to pregnant females from E16 to P0 in order to activate transgene expression from inducible constructs.

### Molecular constructs

EPOR shRNA plasmid (shEPOR) was ordered from Santa Cruz Biotechnology (sc-77364-SH). The original product was a pool of three target-specific lentiviral vector plasmids each encoding 19-25 nucleotides (plus hairpin) designed to knockdown EPOR gene expression. Each of these plasmids constitutively co-expressed shEPOR with GFP. In order to minimize the volume of the original pool of shEPOR plasmids, the three plasmids were separated and their efficacy was tested *in vivo* through *in utero* electroporation. Two plasmids out of the three had a significant effect on cellular position [percentage of electroporated cells in layer IV at P0: GFP (control), 78.84%; plasmid 1, 78.23%; plasmid 2, 56.63%; plasmid 3, 38.60%]. The most efficient shEPOR plasmid was selected and was modified as explained below. EPO shRNA plasmid (shEPO) was similarly ordered from Santa Cruz Biotechnology (sc-270111-SH). Plasmids for the expression of EPO, EPOR, Akt and ERK::MEK were all modified in our lentivector platform, which is used as a mammalian expression system, using recombination cloning based on the Gateway system (Giry-Laterrière et al., 2011). Genes were first PCR-cloned from various templates (Table S1), sequenced and then incorporated into lentivector backbones containing a GFP expression cassette for the live tracking of transduced cells. Some genes were also cloned into all-in-one auto-inducible lentivectors containing our optimized Tet-On system and GFP for live tracking (Giry-Laterrière et al., 2011). Expression plasmid for EKAR [nuclear EKAR (EGFP-mRFP)] was directly obtained from Addgene (#18682) (see Table S1). Plasmids for expression of microRNA to downregulate specific genes were also constructed using our lentivector expression system. In-house microRNA design (hereinafter called mirGE) was used because of its unparalleled efficiency compared with other microRNA design (Myburgh et al., 2014). The mirGE hairpins (see Table S2) were cloned as tandem repeats of three loops downstream of a spacer composed of the GFP open reading frame in which three stop codons had been inserted to prevent translation of the GFP protein (deltaGFP). These hairpins were then cloned by Gateway cloning into lentivectors expressing GFP or auto-inducible lentivectors containing our optimized Tet-On system and either GFP or blasticidin S deaminase for live tracking or selection (Giry-Laterrière et al., 2011). See Giry-Laterrière et al. (2011) for more details about the promotor efficiency and the timecourse of DOX activation. In addition, more details about expression plasmids are provided in Table S1, and full maps and sequences are available from Addgene (www.addgene.org) and our institutional website (lentilab.unige.ch).

### *In utero* electroporation

*In utero* electroporation was performed on Wistar rats on E16 in order to target future layer IV precursors as described previously (Petrenko et al., 2015; Saito and Nakatsuji, 2001). Pregnant Wistar rats at E16 were anesthetized with 1.5% isoflurane in a mixture of 30% O_2_ and 70% air. The abdomen of the animal was opened by way of a midline laparotomy and the right uterus horn was exposed through the operating wound. Subsequently, 2 μl of plasmid was injected into the left lateral ventricle of the developing brain. For control animals, GFP-overexpressing plasmid at 0.5 μg/μl was used. For experimental animals, the following plasmids were used:, shEPOR- and ishEPOR-overexpressing plasmids at 2 μg/μl+GFP-overexpressing plasmid at 0.2 μg/μl; iEPO-overexpressing plasmid at 5 μg/μl+GFP-overexpressing plasmid at 0.5 μg/μl; Akt-overexpressing plasmid at 3 μg/μl+GFP-overexpressing plasmid at 0.3 μg/μl; ERK-overexpressing plasmid at 1 or 3 μg/μl+GFP-overexpressing plasmid at 0.1 or 0.3 μg/μl; iERK-overexpressing plasmid at 1 μg/μl+GFP-overexpressing plasmid at 0.1 μg/μl; ishEPO-overexpressing plasmid at 4 μg/μl+GFP-overexpressing plasmid at 0.4 μg/μl; and EKAR-overexpressing plasmids at 2 μg/μl. To exclude off-target effects, we used an shRNA targeting an mRNA sequence unrelated to EPOR, namely the mRNA sequence of the chemokine receptor 5 (CCR5). This receptor has no known functions in neuronal migration. Cell positioning analysis did not reveal any significant differences in cell positioning between cells electroporated with the ‘off-target’ shRNA and the GFP-overexpressing plasmid used as a control in this study (2C). For electroporation, 0.5 cm diameter tweezers with round platinum plate electrodes (NepaGene, CUY650P5) were positioned on the head of the fetus with the positive electrode positioned over the skull covering the region of the dorsolateral somatosensory cortex (ipsilateral to the injection site). This procedure allowed us to target the future layer IV neuronal precursor cells located in the dorsolateral ventricular zone. Each pup received five 50 ms electrical pulses of 50 V at a rate of 1 pulse/s using the CUY21 SC Square Wave Electroporator (NepaGene). During the entire procedure, pups were hydrated with PBS. The uterus horn was then reintroduced into the abdominal cavity and the same procedure was repeated for the second horn. Finally, the abdominal cavity was sutured using uninterrupted stitches for the peritoneum and individual stitches for the skin with polypropylene monofilament fiber (4-0 or 5-0, Ethicon).

### HEK 293T/17 cell cultures and plasmid transfection

For the validation of the genetic constructs, the following *in vitro* system was used. HEK 293T/17 cells were plated on poly-L-lysine-coated six-well plates at a density of 400,000 cells/well and maintained in culture at 37°C with 5% CO_2_ in Dulbecco's Modified Eagle Medium (Sigma-Aldrich) containing 10% fetal calf serum and 1% penicillin/streptomycin. After 24 h, cells were transfected using the jetPEI technique (Polyplus transfection). For shEPOR and ishEPOR validation, control cells were transfected with a plasmid that constitutively expressed EPOR and experimental cells were transfected with the same EPOR-overexpressing plasmid plus shEPOR (Fig. S1B) or ishEPOR (Fig. S4A). The overexpression of EPOR in control cells was needed because HEK 293T/17 cells express only a negligible amount of EPOR, which was not enough to test the function of our shEPOR or ishEPOR plasmids (see the GFP group in Fig. S1B). For iEPO validation, HEK 293T/17 cells were transfected with iEPO (Fig. S7C). For ishEPO validation, controls cells were transfected with a plasmid that constitutively expressed EPO and experimental cells were transfected with the same EPO-overexpressing plasmid plus ishEPO (Fig. S7E). The overexpression of EPO in control cells was needed because HEK 293T/17 cells express only a negligible amount of EPO, which was not enough to test the function of our ishEPO plasmid (see the GFP group in Fig. S7E). DOX was added to the medium at a concentration of 1 mg/ml immediately after transfection for ishEPOR, ishEPO and iEPO validation. Transfected cells were collected for RNA extraction 72 h after transfection.

### *In situ* hybridization

Frozen sections (40 μm) were melted on SuperFrost Ultra Plus slides (Menzel-Gläser), dried at room temperature (RT) for 1 h and fixed for 10 min in 4% paraformaldehyde (PFA, Sigma-Aldrich)/PBS (Sigma-Aldrich) at RT. After washing in PBS containing 0.1% Tween 20 (PBST, Sigma-Aldrich), slides were digested with 2 μg/ml proteinase K (Roche) at RT for 40 s, then washed again in PBST, and treated with 2 mg/ml glycine (Biosolve) in PBST for 5 min at RT. Following washing in PBST, sections were fixed again for 10 min in 4% PFA/PBS at RT, and were washed in PBST. Sections were then prehybridized in a solution containing 50% deionized formamide (Sigma-Aldrich), 0.2 M NaCl, 10 mM Tris, 5 mM sodium dihydrogen orthophosphate, 5 mM sodium phosphate dehydrate, 50 mM EDTA (AppliChem), 10% dextran sulfate (Sigma-Aldrich), 10% salmon sperm (Ambion) and 1× Denhardt's solution (Sigma-Aldrich) for 1 h at 60°C. All solutions used before probe hybridization were treated with 1% diethylpyrocarbonate (DEPC, Carl Roth) and autoclaved. Prehybridization steps were carried out under RNase-free conditions. Overnight (ON) hybridization was carried out in a humidified chamber (hybridization oven, IN55plus, Memmert) at 60°C with digoxigenin-labeled cRNA probes. Hybridized probes were incubated with an anti-digoxigenin antibody (Roche, ab420) diluted 1/2000 in alkaline phosphatase (AP, Roche) ON at 4°C. AP was detected using nitro blue tetrazolium/5-bromo-4-chloro-3-indolyl phosphate solution (Roche) with reaction times ranging from 3 to 5 days. Slices were fixed in 4% PFA/PBS for 30 min, rinsed in PBST and mounted using an aqueous medium (Aquatex, VWR). Negative control slides were digested with RNase A (60 min at 37°C, Carl Roth) before hybridization.

### RNA analysis

Gene expression was assessed by qRT-PCR. First, RNA was extracted using an RNeasy kit (Qiagen, 74104) and converted into cDNA using SuperScript II Reverse Transcriptase (Invitrogen, 18064014). The cDNAs were then used as template for qPCR using primers specific for rat EPO and EPOR. Primers for cyclophilin A were used as internal controls to normalize for total mRNA content (see Table S3 for primer list). qPCR was performed using SYBR Green reagent (Roche, 4913850001) and an Applied Biosystems 7500 Thermal Cycler. Final results are expressed as deltaCT of specific gene/control gene.

*In situ* RNA analysis was performed using riboprobes specific for rat EPO and EPOR. A DNA template was generated by PCR using Herculase II DNA polymerase (Agilent, 600675) on cDNA template obtained from P0 rat brain RNA, and sequence-specific primers containing either SP6 or T7 sequence (see Table S3 for primer list). Antisense specific probes were then generated by transcription from the PCR DNA template with the T7 RNA polymerase (Roche, 10881767001). Labeling of T7 antisense probes was performed using a DIG-RNA labeling mix (Roche, 11277073910) with DIG as the label on the UTP nucleotide. Staining was then performed by incubation with a sheep anti-DIG-AP Fab fragment (Roche, 11 093 274 910), followed by incubation with NBT/BCIP mix (Roche, 11681 451001). Negative controls were performed with samples incubated with ribonuclease A (Carl Roth, C7156.1) before incubation with T7 antisense probes.

### Cortical slices preparation for time-lapse confocal imaging

Real-time imaging of migrating neurons was carried out according to previously published methods (Boitard et al., 2015; Dayer et al., 2007; Zgraggen et al., 2012). To extract E19/E21 pups, the mother was anesthetized with 1.5% isoflurane in a mixture of 30% O_2_ and 70% air. Extracted brains of E19/E21 pups were embedded in Hank's balanced salt solution and 4% agarose. Afterwards, 300 μm coronal slices of the obtained brains were cut on a vibratome (Vibratome Company) and were then placed on porous nitrocellulose inserts (Millicell-CM, Millipore) in a 35-mm FluoroDish culture dish (World Precision Instruments) containing 1 ml of Neurobasal medium (Gibco by Invitrogen) supplemented with 2% B27, 200 mM L-glutamine, 1 M N-acetylcysteine, 1 M Na-pyruvate and 1% penicillin and streptomycin that was stored in a thermoregulated incubator (37°C, 5% CO_2_) for 6 h. Long-term imaging was performed by time-lapse sequence recordings with a Nikon A1R Resonant Scanner Upright Microscope maintained in a thermoregulated chamber (37°C, 5% CO_2_) for 12 to 14 h (one large image/10 min) using a 488 nm laser illumination diode, 20× Plan Fluor ELWD DIC L objective and NIS-Elements AR software (v4.30.02). Multi-dimensional acquisitions were performed using the Perfect Focus System to decrease focus drift and allow the acquisition of large mosaic images obtained from the stitching of nine adjacent fields, produced by the stacking of 21 consecutive slides with a 5 μm space inbetween.

### Tissue processing for post-hoc *in vivo* studies

For post-hoc *in vivo* studies, rat brains were analyzed at different time points: E16, E17, E19, P0, P2, P5, P7, P21 and P35. To extract E16, E17 and E19 pups, the mother was anesthetized with 1.5% isoflurane in a mixture of 30% O_2_ and 70% air. P0, P2 and P5 animals were sacrificed by rapid decapitation. The extracted brains of E16, E17, E19, P0, P2 and P5 pups were directly fixed in 4% PFA at 4°C. After 1 day of fixation, the brains were washed in PBS and then cryoprotected with 10% sucrose for 24 h and 20% sucrose for another 24 h, before 20 μm cryostat sections were cut. P7, P21 and P35 animals were anesthetized with pentobarbital (Streuli Pharma) and sacrificed by intracardial perfusion of 0.9% saline followed by 4% PFA (Biochemica). Brains were postfixed ON in 4% PFA at 4°C. After postfixation for 24 h, the brains were washed with PBS before 50 μm vibratome slices were cut.

### Immunohistochemistry

Immunofluorescence was carried out according to protocols described previously (Zgraggen et al., 2012). Previously prepared slices were washed three times with 0.1 M PBS. Slices were pre-incubated with PBS-bovine serum albumin (BSA)-Triton X-100 (TX) buffer (0.5% BSA, 0.3% Triton X-100 and 0.1% NaN3) for 60 min at RT in the dark. Subsequently, cells were incubated with primary antibodies diluted in PBS-BSA-TX ON at 4°C in the dark. The following day (or 2 days later for SATB2 and cFos) cells were washed three times with 0.1 M PBS and incubated for 1 h 30 min at RT in the dark, with the secondary antibodies diluted in PBS-BSA buffer (0.5% BSA and 0.1% NaN3). After three washes with 0.1 M PBS, cell nuclei were counterstained with Hoechst 33342 (Invitrogen, H3570) (Invitrogen) diluted 1/5000 in 0.1 M PBS. Finally, coverslips were mounted using Immu-Mount (Thermo Scientific). In this study, the following primary antibodies were used: polyclonal goat anti-GFP (1/200, LubioScience, NB100-1770); polyclonal rabbit anti-EPOR (1/500, Sigma-Aldrich, SAB4500780); polyclonal mouse anti-nestin (1/1000, Millipore, MAB353); polyclonal rabbit anti-TBR2 (1/500, Abcam, AB23345); monoclonal mouse anti-SATB2 (1/250, Abcam, SATBA4B10); polyclonal rabbit anti-Ki67p (1/1000, Novacastra, AA1250-1300); monoclonal mouse anti-NeuN (1/500, Millipore, MAB377); polyclonal rabbit anti-CUX1 (1/500, Santa Cruz Biotechnology, SC13024); polyclonal rabbit anti-CTIP2 (1/300, Abcam); polyclonal rabbit cleaved caspase 3 (1/200, Cell Signaling, AB2302); and polyclonal rabbit anti-cFos (1/100, Oncogene, 226003). The following secondary antibodies were used at a dilution of 1/1000: donkey anti-goat 488 (Alexa Fluor, A32814); donkey anti-mouse 568 and 647 (Alexa Fluor, A10037 and A32787, respectively); and donkey anti-rabbit 568 and 647 (Alexa Fluor, A10042 and A32795, respectively).

### Image acquisition, analysis and quantification

For analyses on post-hoc tissue, immunostained slices were examined using a Zeiss LSM700 confocal laser-scanning microscope with Apo Plan-Neofluar 10× NA 0.30 and Apo Plan-Neofluar 20× NA 0.50 objectives for low-power magnification and, for high-power magnification, Plan-Neofluar 40× NA 1.3 Oil and Plan-Neofluar 63× NA 1.4 Oil objectives were used, as well as 405-nm, 488-nm, 561-nm and 640-nm laser illumination diodes. Large-field images were assembled using Adobe Photoshop. For ISH images, a wide-field slide-scanner Olympus BX61 VS120 microscope with 40× S Apo objective was used.

Measurement of the intensity of EKAR-labeled cells was accomplished using confocal images covering the entire cortex length. For image capture, the intensity of fluorescent excitation of cells, gain and black levels were kept constant for each session of measurement. The pixel intensity threshold for RFP and GFP was adjusted such that the tissue background corresponded to level 0. The ratio of RFP-intensity over GFP-intensity of each cell was then measured using the ImageJ64 software (v1.48). Statistical significance was calculated using one-way ANOVA followed by Bonferroni correction. Results are expressed as mean±s.d.; *n*=number of analyzed brains from at least two different experiments. For each region, three slices were analyzed.

Quantification of SATB2 or TBR2 staining of GFP-labeled cells at E19 was accomplished using confocal images covering the SVZ and IZ. Quantification of cleaved caspase 3 (CC3) or Ki67p staining of GFP-labeled cells at E17 and E19 was accomplished using confocal images covering the VZ/SVZ, IZ and CP. Quantification of EPOR, NeuN, Cux1, CTIP2 and cFos staining at P21 or P35 was accomplished on confocal images covering layer III or IV for experimental and control conditions, or the rest of the cortex for misplaced cells. A set of GFP (control) and shEPOR (or ishEPOR) electroporated brain slices were immunostained against EPOR, SATB2, TBR2, CC3, Ki67p, NeuN, Cux1 or CTIP2 and analyzed simultaneously. P35 GFP (control), ishEPOR and iEPO electroporated brain slices were immunostained against NeuN and cFos and analyzed simultaneously. NeuN and cFos immunostaining and analysis of the ishEPOR+iERK condition was realized at a later time point and compared with previously obtained results. In total, three slides (with three pictures per slide and per region) per brain were analyzed. Statistical significance was calculated using unpaired two-tailed Student's *t*-test. Results are expressed as mean±s.d.; *n*=number of analyzed brains coming from at least two different experiments.

For cell positioning/bin analysis, sections from E16 or E18 electroporated brains were used. Using MetaMorph software, cell coordinates were projected on the closest curve following the path of radial glia fiber to estimate the relative percentage of migration progress. VZ and SVZ were not included in the migratory path. The fraction of migrating cells for each 5% progress step was defined as bin 1 to 20 (1 for basal, 20 for apical). GFP (control), shEPOR, ishEPOR (no DOX) and ishEPOR (+ DOX) conditions were electroporated and analyzed simultaneously. The ishEPO (+ DOX), iEPO (+ DOX) and iEPORT (+ DOX) were electroporated and analyzed at the same time as their respective no DOX conditions (direct control), and were compared with the previously analyzed GFP group. ERK and Akt conditions were electroporated and analyzed at the same time, and compared with the previously analyzed GFP group. Rescue experiments were realized at a later time point and compared with previously obtained results. For each brain, three slices were analyzed. Statistical significance was calculated using two-way ANOVA followed by Bonferroni correction. Results are expressed as mean±s.e.m.; *n*=number of analyzed brains coming from at least two different experiments.

The distance between GFP-labeled cells leading process and nestin-labeled radial glia in the CP was measured using MetaMorph software, starting at the base of the leading process. GFP (control) and ishEPOR conditions were electroporated, immunostained and analyzed simultaneously. Rescue experiments were realized at a later time point and compared with previously obtained results. Statistical significance was calculated using two-way ANOVA followed by Bonferroni correction. Results are expressed as mean±s.e.m.; *n*=number of analyzed brains coming from at least two different experiments.

For video time-lapse analyses, slight drifts correction of the slices and single cell tracking were performed with ImageJ64 Software (v1.48). A set (1 or 2 brains) of GFP (control) condition were electroporated and analyzed simultaneously to shEPOR, ishEPOR, ishEPO and iEPO conditions. Rescue experiments were realized at a later time point and compared with previously obtained results. Results are presented as mean±s.e.m. and *n* (number of analyzed brains with 30 tracked cells per brain) was at least four, from two different experiments. In these analyses, multipolar cells were cells that displayed multipolar morphology (at least three primary processes) at least once during tracking time, immobile cells were cells that did not move at all during tracking time, inverted cells were cells that exhibited a complete inversion of their migrating direction at least once during tracking time, and IZ-exiting cells were cells that were present in the upper IZ at the beginning of the tracking and were able to enter the CP. Statistical significance was calculated using an unpaired *t*-test or one-way ANOVA, followed by Bonferroni correction. Results are expressed as mean±s.d.; *n*=number of analyzed brains from at least two different experiments.

Neurolucida software was used to create morphological reconstruction using ten cells per layer in each brain. GFP (control) and ishEPOR conditions were electroporated, immunostained and analyzed simultaneously. Rescue experiments were realized at a later time point and compared with previously obtained results. Statistical significance was calculated using an unpaired *t*-test or one-way ANOVA followed by Bonferroni correction. Results are expressed as mean±s.d.; *n*=number of analyzed brains coming from at least two different experiments.

For dendritic protrusion analysis, the part of a second-order dendrite situated immediately after the first dendritic branching was imaged over its entire height at high-power magnification and protrusions were then counted as described previously (Holtmaat et al., 2009), taking two dendrites per cell and ten cells per region of the brain. GFP (control) and ishEPOR conditions were electroporated, immunostained and analyzed simultaneously. Rescue experiments were realized at a later time point and compared with previously obtained results. Statistical significance was calculated using an unpaired *t*-test or one-way ANOVA followed by Bonferroni correction. Results are expressed as mean±s.d.; *n*=number of analyzed brains from at least two different experiments.

### Recording of somatosensory evoked potentials (SEPs)

We recorded intracortical local field potentials in P32-P40 GFP and ishEPOR Wistar rats under light isoflurane anesthesia (recordings at 0.8-1.1% in 30%O_2_/70% air) as described previously (Plomp et al., 2014). Linear 16-electrode probes (177 μm^2^ electrode diameter, 100 μm inter-electrode spacing, NeuroNexus Technologies) were inserted perpendicular to the cortical surface in the middle of the large whisker representations of the S1 barrel cortex regions of both hemispheres (1.5 mm posterior and 5 mm lateral to bregma). Probes were painted with a fluorescent marker (Dye I, Invitrogen) to recover their positions via histology. Differential potentials against a skull reference electrode were acquired with the multichannel Digilynx system (Neuralynx). The LFP signal was bandpass-filtered online between 1 and 4000 Hz and sampled at 8 kHz. Whisker stimuli (500 µm back-and-forth deflections with 1 ms rise time) were delivered by solenoid-based stimulator devices simultaneously to all large whiskers on one side of the face (100 right-sided and 100 left-sided stimuli in randomized order with 9 s inter-stimulus intervals).

Recordings were further filtered offline for LFP analyses (1-250 bandpass). SEPs were averaged around 100 ms pre-stimulus to 500 ms poststimulus. One-dimensional current-source density (CSD) were calculated as the product of the second spatial derivative of the electric potentials along the cortical depth (conductivity tensor assumed to be constant) using the following formula: CSD=−[V(z+Δz, t)−2V(z, t)+V(z−Δz,t)]/Δz2; where V(z, t)=measured voltage at subpial depth z, t=time and Δz=100 µm (Mitzdorf, 1985). Analyses were performed using Cartool software by Denis Brunet (cartoolcommunity.unige.ch), and MATLAB toolboxes (MathWorks).

### Sensory-motor behavior

A battery of sensory-motor behavior tasks was performed on female and male P7 and P35 rats. GFP (control), ishEPOR and iEPO conditions were electroporated and tested simultaneously. Rescue experiments were realized at a later time point and compared with previously obtained results. After behavior, the phenotype was anatomically and morphologically checked.

### Neonatal behavior

#### Vibrissae-evoked behavior

Neonatal motor response to sensory stimulations was examined in P7 pups, after the development of the barrel cortex (Rice, 1985). Pups were removed from the mother and placed in the center of a sagex arena (15×15 cm) under a thermoregulatory lamp. A 5 min acclimation period preceded the experimental session to allow recuperation from experimental handling. Unilateral whisker stimulation was executed manually using a 1 mm wooden rod (one stimulation was considered a sweep back or forth across the entire whisker field). Two trials per whisker-side were performed, in each of which 30 stimulations were carried out and the number of motor responses was observed. Finally, the average of the trials was calculated and the results of the contralateral side were compared with the ipsilateral side (control) with a paired two-tailed Student's *t*-test. Results are expressed as mean±s.e.m.; *n*=number of animals from at least two different experiments.

#### Young-adult behavior

During the period of habituation (14 days before the battery of tests), animals were placed in the testing environment for 30 min per day and were handled by the experimenters. All habituation phases and tests were performed during the first 6 h of the light phase of the 24-h light/dark cycle.

### Gap-crossing test

Whisker-specific tactile abilities were examined in animals using the gap-crossing test. The gap-crossing testing arena was composed of a 40×40 cm platform that was elevated from the floor by 100 cm, with a central adjustable wide gap separating the two sides of the chamber: one side brightly illuminated and the other one obscured and containing nesting material from the home cage. At P35, rats were placed in the bright side of the platform, and the nature of thigmotaxis would drive the animal to move into the dark side, forcing it to cross the gap. The gap-crossing procedure was conducted in a series of 1 cm increasing gap distances (ranging from 6 cm to a maximum of 16 cm), and for each of the two allowed trials the time to cross and the number of whisking-trials before each crossing were measured. If the rat was not able to cross the gap or stayed in the bright side of the platform for more than 60 s, the trial was considered failed. Uncrossed distance was determined when the rat failed two consecutive trials for the same distance. Statistical significance was calculated using two-way ANOVA followed by Bonferroni correction. Results are expressed as mean±s.e.m.; *n*=number of animals coming from at least two different experiments.

### Adhesive patch detecting test

To assess somatosensory touch sensation and complex sensorimotor functions, we used the adhesive patch removal task. A 6-mm diameter circular adhesive patch was placed on the plantar surface of one hindpaw, after which rats were released in the testing arena and observed for a maximum of 240 s. Latency to detect the patch (first snout contact with the patch) was measured. Rats randomly underwent four consecutive trials (two where the patch was placed on the contralateral hindpaw, and two where the patch was placed on the ipsilateral hindpaw). Finally, the average of the trials was calculated per side and the results of the contralateral hindpaw were compared with the ipsilateral hindpaw (control) with a paired two-tailed Student's *t*-test. Results are expressed as mean±s.e.m.; *n*=number of animals coming from at least two different experiments. Time to complete the removal of the patch was also measured (if the animal did not remove the patch in the 240 s allowed time, the patch was removed by the experimenter and time was counted as 240 s). The average of the trials was calculated for each side and the results of the contralateral hindpaw were compared with the ipsilateral hindpaw (control) with a log-rank test. If no difference was found between sides, all trials were averaged together and experimental animals were compared with control ones with a log-rank test.

### Enriched environment

At the beginning of the dark part of the light/dark cycle, P35 animals were left in the dark for 80 min inside an unknown 34×60×27 cm cage containing several unknown materials that were changed at 30 min and 60 min timepoints. Animals were then immediately perfused and tissue was processed for analysis.

### Data analyses

Statistical analysis was conducted using GraphPad Prism 7. The normality of sample distributions was assessed with the Shapiro–Wilk criterion. Data are represented as the mean±s.d. or ±s.e.m. (see figure legends) and the significance was set at 95% of confidence. Statistical significance was assessed using a paired or unpaired two-tailed Student's *t*-test between two groups. One-way or two-way ANOVA, followed by a Bonferroni multiple comparisons post-test, was used to compare multiple groups. Statistical significance was defined as *P*<0.05 (*), *P*<0.01 (**), *P*<0.001 (***) and *P*<0.0001 (****).

## Supplementary Material

Supplementary information

Reviewer comments
