# Supplementary material for: Endogenous erythropoietin signaling regulates migration and laminar positioning of upper-layer neurons in the developing neocortex
Source: Development. 2020 Oct 7;147(19):dev190249. doi: 10.1242/dev.190249 (PMC7561482; doi:10.1242/dev.190249)
Supplement: Supplementary information [file develop-147-190249-s1.pdf]

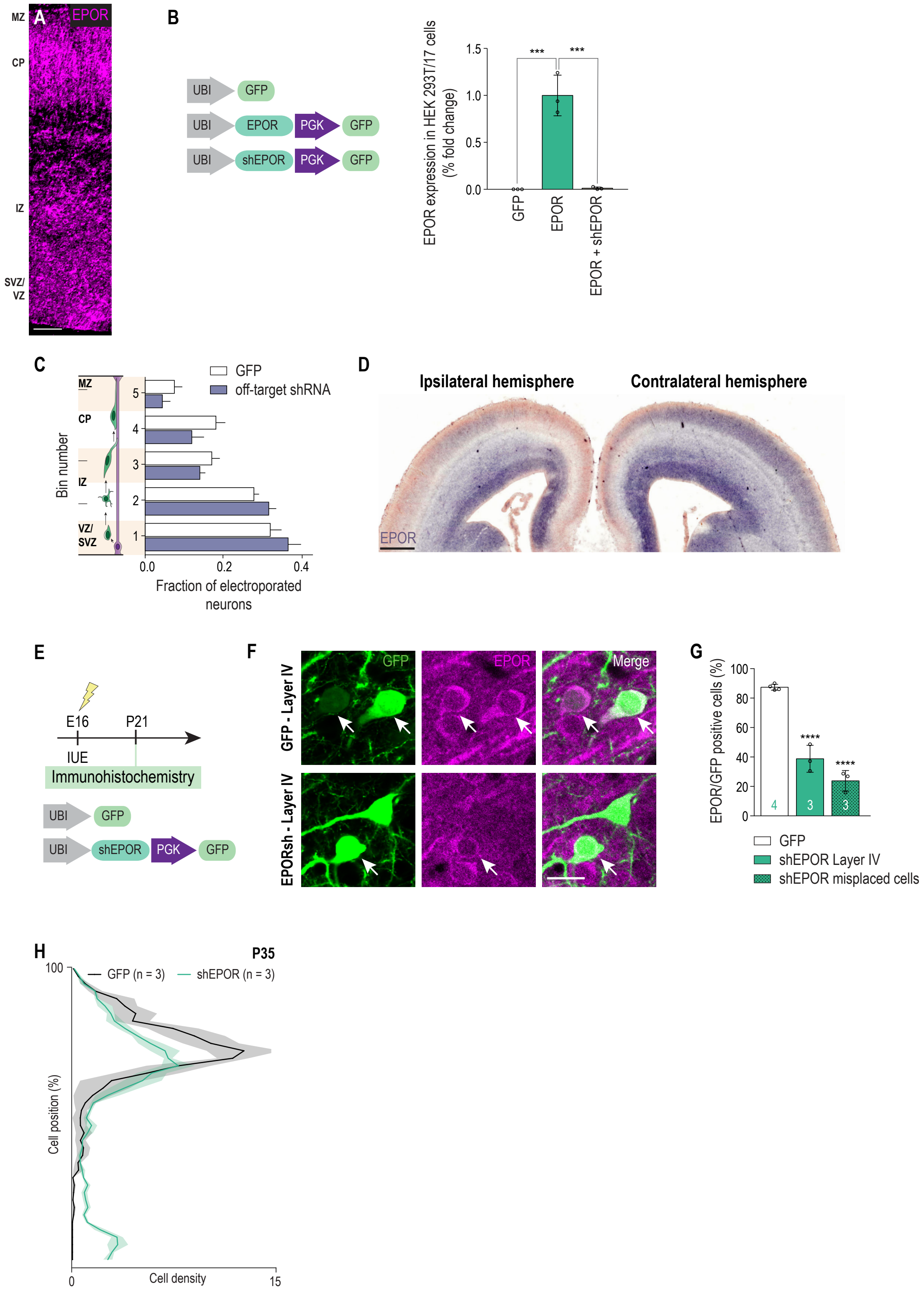

Development • Supplementary information

Figure S1: Validation of shEPOR plasmid.

(A) Immunostaining against EPOR on E19 cortical slices. **(B)** Relative EPOR expression in HEK 293T/17 cells transfected with GFP, EPOR and EPOR + shEPOR (GFP: *n*=3, EPOR: *n*=3, EPOR + shEPOR: *n*=3. One-way ANOVA followed by Bonferroni's multiple comparisons test: $F_{(2, 6)}$ = 63.17, $P<0.0001$). **(C)** Cell positioning analysis of an E18 electroporated off-target shRNA construct analyzed at P3 (GFP: *n*=4, off-target shRNA: *n*=3.) showed no statistically significant difference in the positioning of shRNA-treated or GFP-electroporated cells. **(D)** Picture of a representative ISH against EPOR at E19 of an E16 shEPOR-electroporated brain showing a decreased staining in the side ipsilateral to electroporation. **(E)** Experimental time-line. **(F)** EPOR immunostaining on control (upper panel) and shEPOR- (lower panel) electroporated cells at P21. **(G)** Percentage of electroporated cells positive for EPOR staining in Layer IV control, Layer IV and misplaced shEPOR conditions (GFP: *n*=4, shEPOR: *n*=3. One-way ANOVA followed by Bonferroni's multiple comparisons test: $F_{(2, 7)}$ = 99.76, $P<0.0001$). **(H)** Quantification of the distribution of electroporated cells at P35 (GFP: *n*=3, shEPOR: *n*=3. Two-way ANOVA followed by Bonferroni's multiple comparisons test: cell position x group interaction: $F_{(39, 156)}=2.607$, $P<0.0001$; cell position main effect: $F_{(39, 156)}=16.74$, $p<0.0001$; group main effect: $F_{(1, 4)}=1.43$, $p=0.3453$). Scale bars: 10 µm (F), 50 µm (A) and 500 µm (D). Error bars, SD. Abbreviations: IUE, *In utero* electroporation. ***$P<0.001$, ****$P<0.0001$

Development • Supplementary information

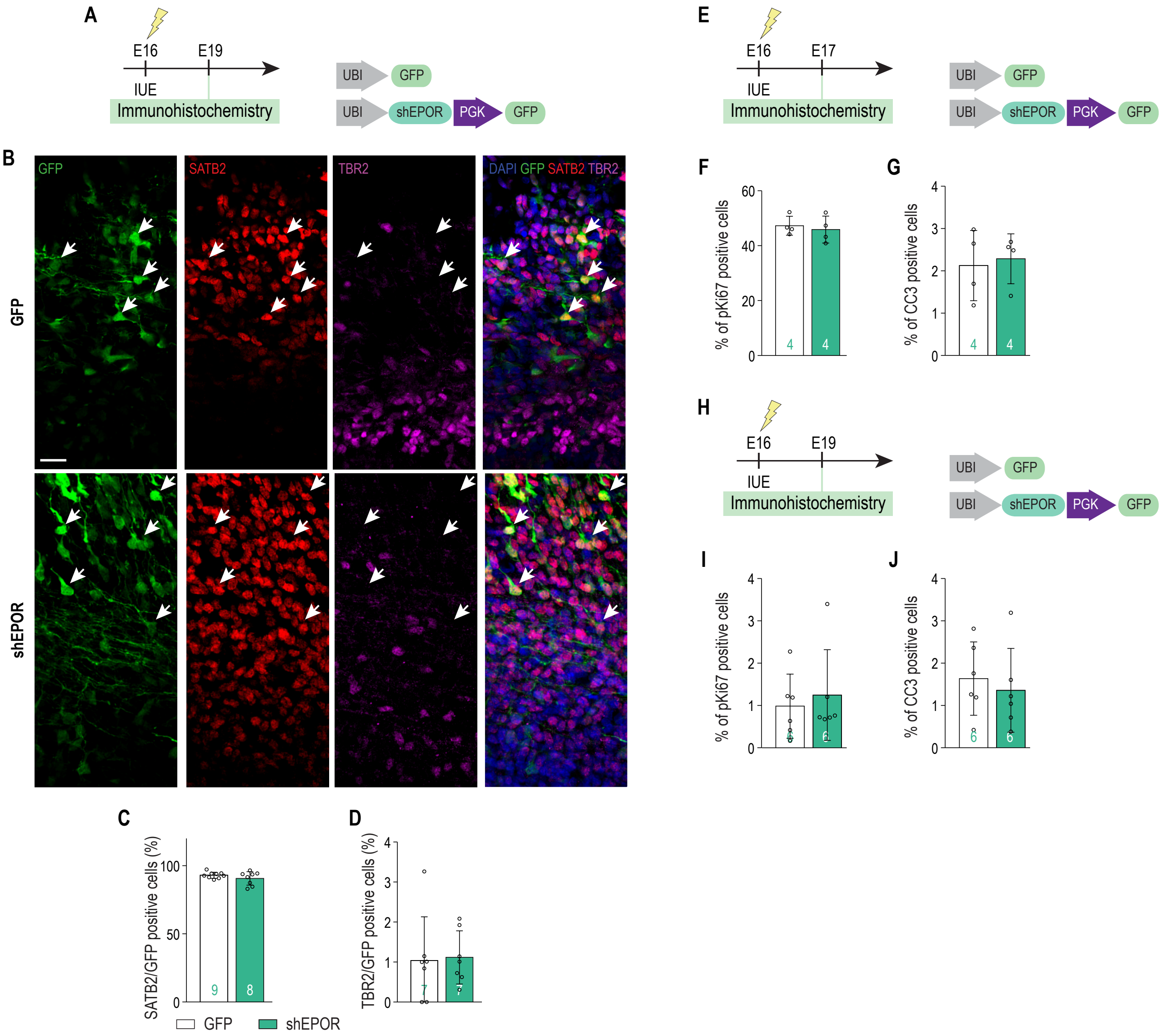

Figure S2: Downregulation of EPOR does not affect early neuronal differentiation, cell cycle and survival.

(A) Experimental time-line. **(B)** SATB2- and TBR2-staining on control (upper panel) and shEPOR- (lower panel) electroporated cells at E19. **(C)** Percentage at E19 of electroporated cells positive for SATB2 staining in control and shEPOR conditions (GFP: n = 9, shEPOR: n = 8. Unpaired t-test: p = 0.2140). (D) Percentage at E19 of electroporated cells positive for TBR2 staining in control and shEPOR conditions (GFP: n = 7, shEPOR: n = 7. Mann-Whitney test: p = 0.6807). **(E)** Experimental time-line. **(F,G)** Percentage at E17 of electroporated cells positive for pKi67- (F) or cleaved caspase 3 (G) staining (n = 4 per condition. Unpaired t-test: p = 0.6576 for F and p = 0.7662 for G). **(H)** Experimental time-line. **(I,J)** Percentage at E19 of electroporated cells positive for pKi67- (F) or cleaved caspase 3 (G) staining (n = 4 per condition. Unpaired t-test: p = 0.6368 for i and p = 0.6185 for j). Error bars, SD. Abbreviations: IUE = *In utero* electroporation.

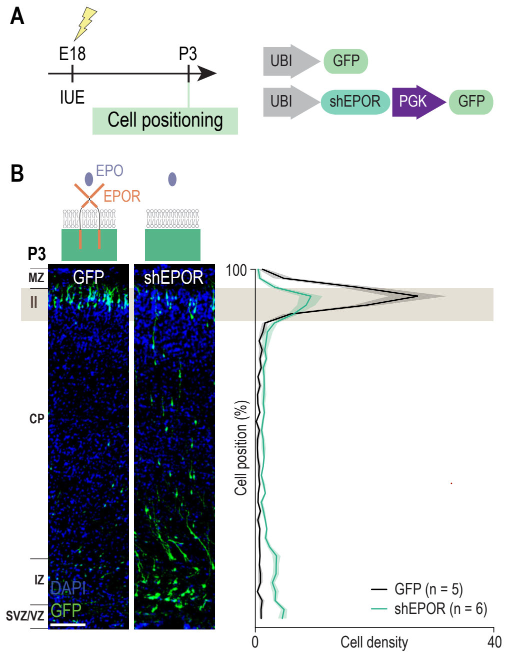

Figure S3: Downregulation of EPOR also affects the neuronal migration of future layer II-III neurons.

(A) Experimental time-line. **(B)** Left: Coronal slices from P3 E18-electroporated brains with GFP or shEPOR. Right: Quantification of the distribution of electroporated cells along the cortex at P3 (GFP: $n = 5$, shEPOR: $n = 6$. Two-way ANOVA followed by Bonferroni's multiple comparisons test: cell position x group interaction: $F_{(39, 351)} = 13.47$, $p < 0.0001$; cell position main effect: $F_{(39, 351)} = 37.18$, $p < 0.0001$; group main effect: $F_{(1, 9)} = 1.227$, $p = 0.2967$). Scale bars : 100 µm (B). Error bars, SD. Abbreviations: IUE = *In utero* electroporation, n = number of analyzed brains (for each brain, more than 200 neurons (B) were considered to define the mean).

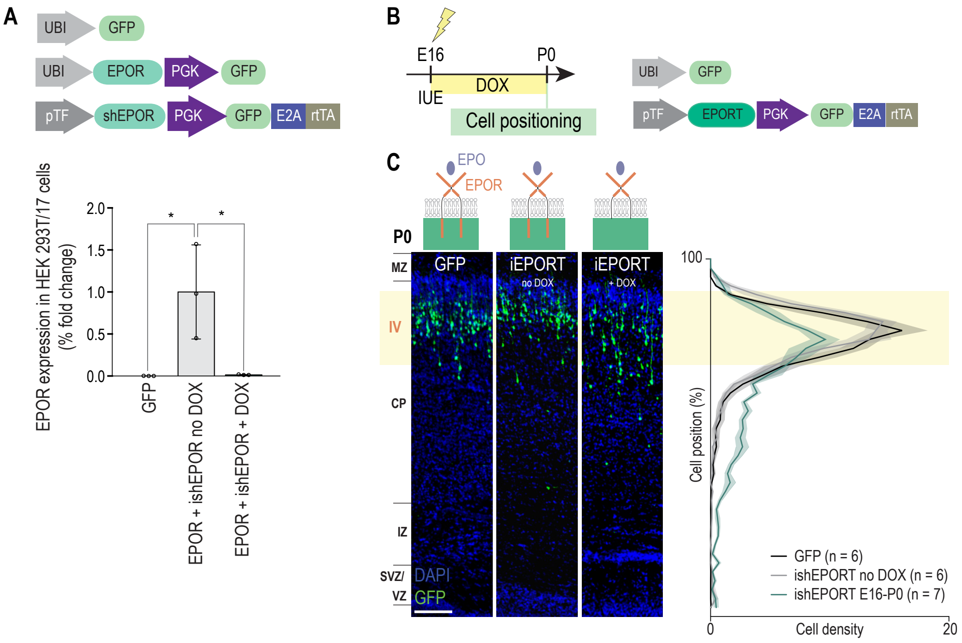

Figure S4: In vitro validation of ishEPOR plasmid and effects on cell migration of the inducible iEPORT plasmid.

(A) Relative EPOR expression in HEK 293T/17 cells transfected with GFP, EPOR + ishEPOR no DOX, EPOR + ishEPOR + DOX (*n* = 3 in each condition. One-way ANOVA followed by Bonferroni's multiple comparisons test: $F_{(2, 6)}$ = 9.433, *p* = 0.0140) **(B)** Experimental time-line. **(C)** Left: Coronal slices from P0 E16-electroporated brains with GFP or a truncated form of EPOR in which we removed its intracellular domain and coupled it to a DOX-inducible promoter (iEPORT). The inducible construct was induced between E16 and P0. Right: Quantification of the distribution of electroporated cells along the cortex at P0 (GFP: *n* = 6, iEPORT no DOX: *n* = 6, iEPORT E16-P0: *n* = 7. Two-way ANOVA followed by Bonferroni's multiple comparisons test: cell position x group interaction: $F_{(78, 624)}$ = 4.743, *p* < 0.0001; cell position main effect: $F_{(39, 624)}$ = 89.72, *p* < 0.0001; group main effect: F $_{(2, 16)}$ = 1.185, *p* = 3311).
The GFP condition shown in C is reported from Fig. 2C. Scale bar : 100 µm (C). Error bars, SD. Abbreviations: IUE = *In utero* electroporation, *n* = number of analyzed brains (for each brain, more than 200 neurons (C) were considered to define the mean), **p*<.05.

Development • Supplementary information

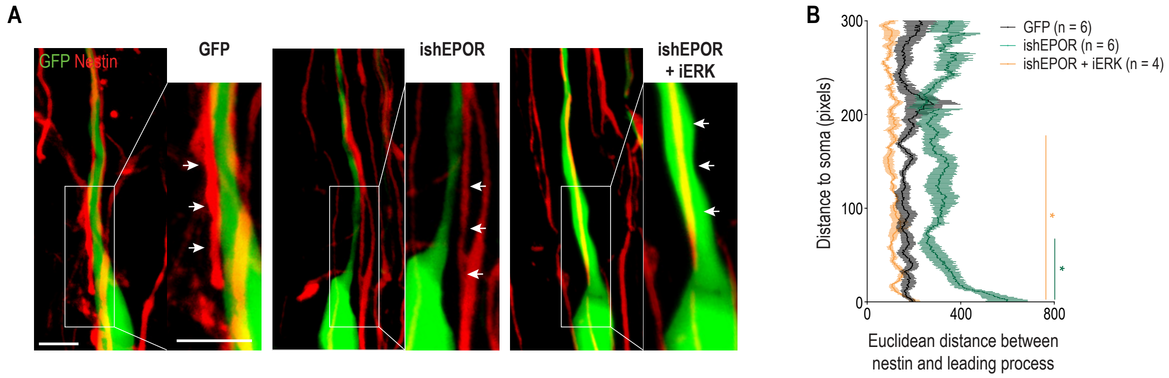

Figure S5: Downregulation of EPOR affects the stability of the neuronal leading process during locomotion. Overexpression of ERK rescues the position of the leading process.

(A) Position of a neuronal leading process (arrows) in respect to nestin-stained radial glia fiber in CP at E19 (GFP: left panel, ishEPOR : middle panel, ishEPOR+iERK : right panel). **(B)** Quantitative analysis of the distance between electroporated cells leading process and the nearest glial fiber (GFP: n = 6, ishEPOR: n = 6, ishEPOR+iERK : n = 4. Two-way ANOVA followed by Bonferroni's multiple comparisons test: distance to soma x distance to nestin: $F_{(798,\ 4662)} = 0.5873$, $p > 0.9999$; distance to soma main effect: $F_{(399,\ 4662)} = 0.8674$, $p = 0.8674$; distance to nestin main effect: $F_{(2,\ 4662)} = 17.67$, $p < 0.0001$). Scale bars : 5 µm (A). Error bars, SD. Abbreviations: n = number of analyzed brains (for each brain more than 50 neurons (B) were considered to define the mean), *p<.05.

Development • Supplementary information

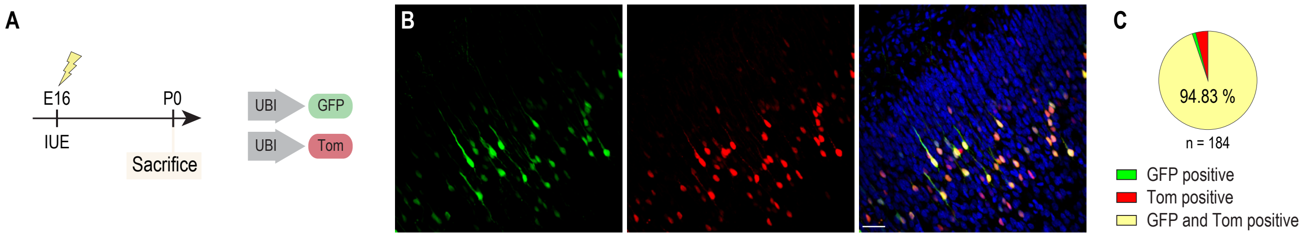

Figure S6: Percentage of double-positive neurons after a co-electroporation

(A) Experimental time-line. We co-electroporated two different plasmids (GFP and Tom). **(B)** Picture taken on P0 brain slices representing the result of the co-electroporation. **(C)** Pie chart reporting the percentage of double-positive neurons in layer IV. Abbreviations: IUE = *In utero* electroporation, *n* = number of counted neurons.

Development • Supplementary information

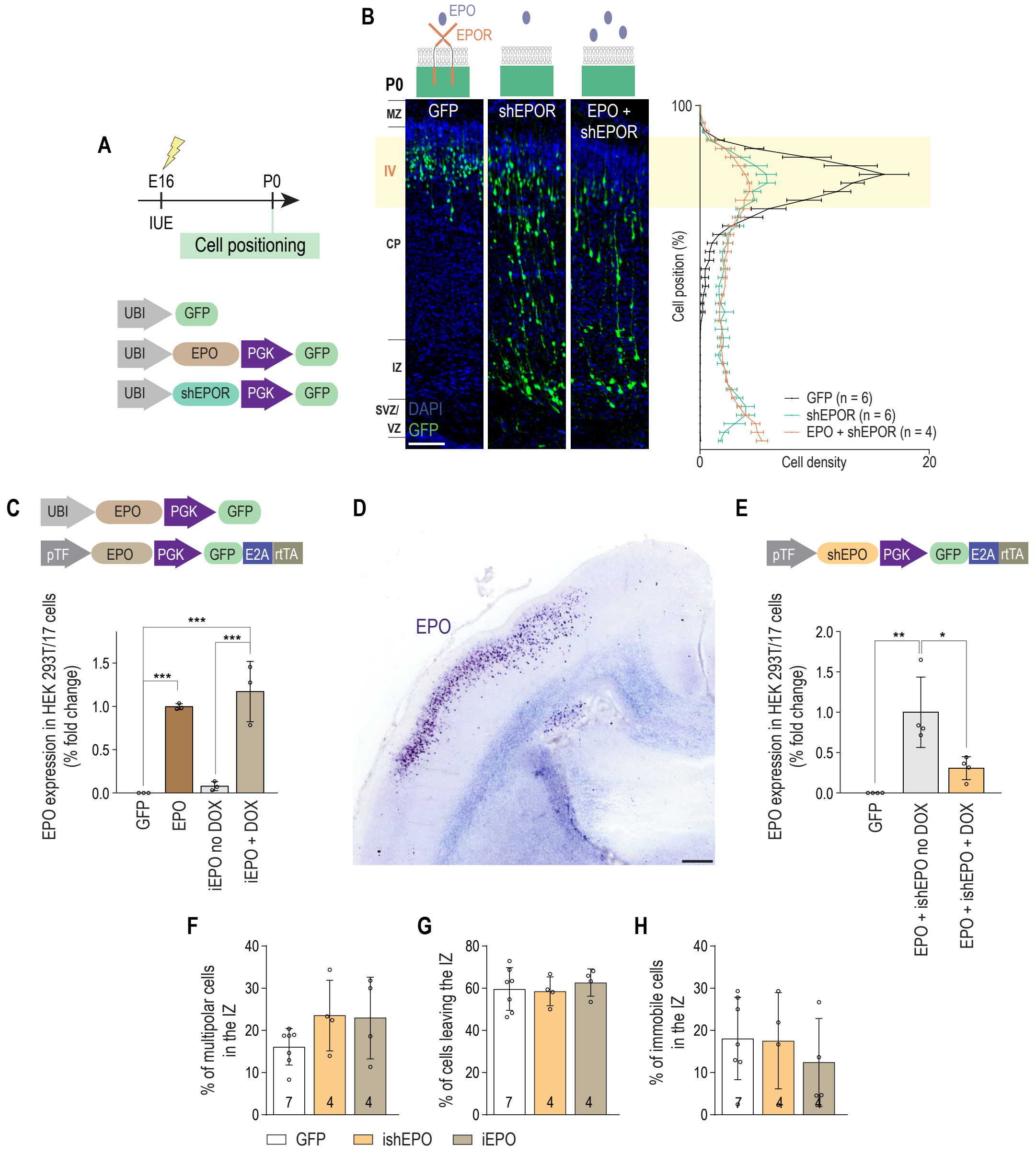
A
E16
P0
IUE
Cell positioning
UBI GFP
UBI EPO PGK GFP
UBI shEPOR PGK GFP
B
EPO
EPOR
P0
MZ
IV
CP
IZ
SVZ/
VZ
GFP
shEPOR
EPO +
shEPOR
DAPI
GFP
Cell position (%)
100
0
Cell density
20
GFP (n = 6)
shEPOR (n = 6)
EPO + shEPOR (n = 4)
C
UBI EPO PGK GFP
pTF EPO PGK GFP E2A rtTA
EPO expression in HEK 293T/17 cells
(% fold change)
0.0
0.5
1.0
1.5
***
GFP
EPO
iEPO no DOX
iEPO + DOX
D
EPO
E
pTF shEPO PGK GFP E2A rtTA
EPO expression in HEK 293T/17 cells
(% fold change)
0.0
0.5
1.0
1.5
2.0
**
*
GFP
EPO + ishEPO no DOX
EPO + ishEPO + DOX
F
% of multipolar cells
in the IZ
G
% of cells leaving the IZ
H
% of immobile cells
in the IZ
7
4
4
GFP
ishEPO
iEPO

Figure S7: In vitro validation of small-hairpin RNA specifically targeting EPO (ishEPOR and iEPO)

(A) Experimental time-line. **(B)** Left: Coronal slices from P0 E16-electroporated brains with GFP, shEPOR and shEPOR + EPO. Right: Quantification of the distribution of electroporated cells along the cortex at P0 (GFP: *n* = 6, shEPOR: *n* = 6, shEPOR + EPO: *n* = 4. Two-way ANOVA followed by Bonferroni's multiple comparisons test: cell position x group interaction: $F_{(78, 507)}$ = 12.01, $p < 0.0001$; cell position main effect: $F_{(39, 507)}$ = 31.6, $p < 0.0001$; group main effect: $F_{(2, 13)}$ = 1, $p = 0.3945$). **(C)** Relative EPO expression in HEK 293T/17 cells transfected with GFP, EPO, iEPO no DOX and iEPO + DOX (*n* = 3 for each condition. One-way ANOVA followed by Bonferroni's multiple comparisons test: $F_{(3, 8)}$ = 35.57, $p < 0.0001$). **(D)** Picture of a representative ISH against EPO at P0 of an E16 iEPO-electroporated brain induced with DOX between E16 and P0 showing a strong staining of the future Layer IV. **(E)** Relative EPO expression in HEK 293T/17 cells transfected with GFP, EPO + ishEPO no DOX, EPO + ishEPO + DOX (*n* = 4 for each condition. One-way ANOVA followed by Bonferroni's multiple comparisons test: $F_{(2, 9)}$ = 14.97, $p = 0.0014$). **(F)** Proportion of cells that exhibited a multipolar morphology during tracking time in the IZ (GFP: *n* = 7, ishEPO: *n* = 4. iEPO: *n* = 4. One-way ANOVA followed by Bonferroni's multiple comparisons test: $F_{(2, 12)}$ = 1.892, $p = 0.1931$). **(G)** Proportion of cells leaving the IZ during tracking time (GFP: *n* = 7, ishEPO: *n* = 4. iEPO: *n* = 4. One-way ANOVA followed by Bonferroni's multiple comparisons test: $F_{(2, 12)}$ = 0.2177, $p = 0.8075$). **(H)** Proportion of tracked cells that remained immobile during tracking time in the IZ (GFP: *n* = 7, ishEPO: *n* = 4. iEPO: *n* = 4. One-way ANOVA followed by Bonferroni's multiple comparisons test: $F_{(2, 12)}$ = 0.1245, $p = 0.8840$). The GFP and shEPOR conditions shown in B are reported from Fig. 2C. Scale bars : 100 µm (B), 250 µm (D). Error bars, SD. Abbreviations: IUE = *In utero* electroporation, *n* = number of analyzed brains (for each brain, more than 50 neurons (F-H) or 200 neurons (B) were considered to define the mean), ***p<.001.

Development • Supplementary information

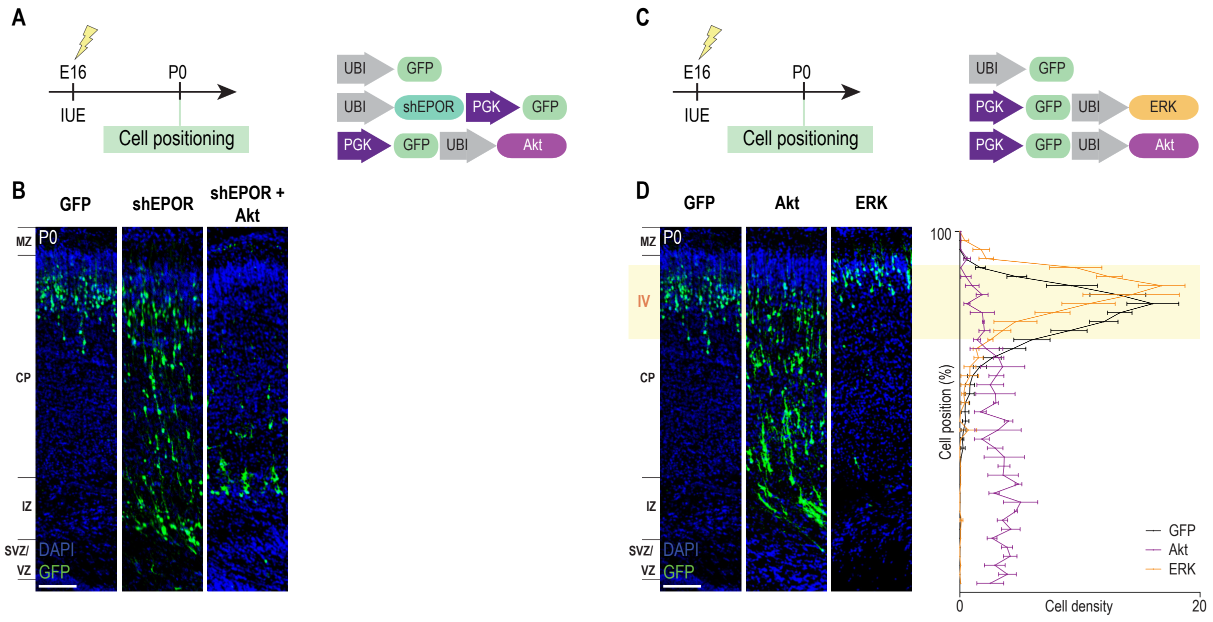

Figure S8. ERK but not AKT upregulation rescues shEPOR-induced migratory deficit.

(A) Experimental time-line. **(B)** Coronal slices from typical P0 brains electroporated at E16 with GFP, shEPOR or shEPOR+Akt. **(C)** Experimental time-line. **(D)** Left: Coronal slices from P0 E16-electroporated brains with GFP, Akt or ERK. Right: Quantification of the distribution of electroporated cells along the cortex at P0 (GFP: *n* = 6, Akt: *n* = 3, ERK: *n* = 3. Two-way ANOVA followed by Bonferroni's multiple comparisons test: cell position x group interaction: $F_{(78, 351)} = 9.949$, $p < 0.0001$; cell position main effect: $F_{(39, 351)} = 20.71$, $p < 0.0001$; group main effect: $F_{(2, 9)} = 0.6$, $p = 0.5694$). The GFP and shEPOR conditions shown in B and D are reported from Fig. 2C. Scale bars : 100 µm (B and D). Error bars, SD. Abbreviations: IUE = *In utero* electroporation, *n* = number of analyzed brains (for each brain, more than 200 neurons (D) were considered to define the mean).

Development • Supplementary information

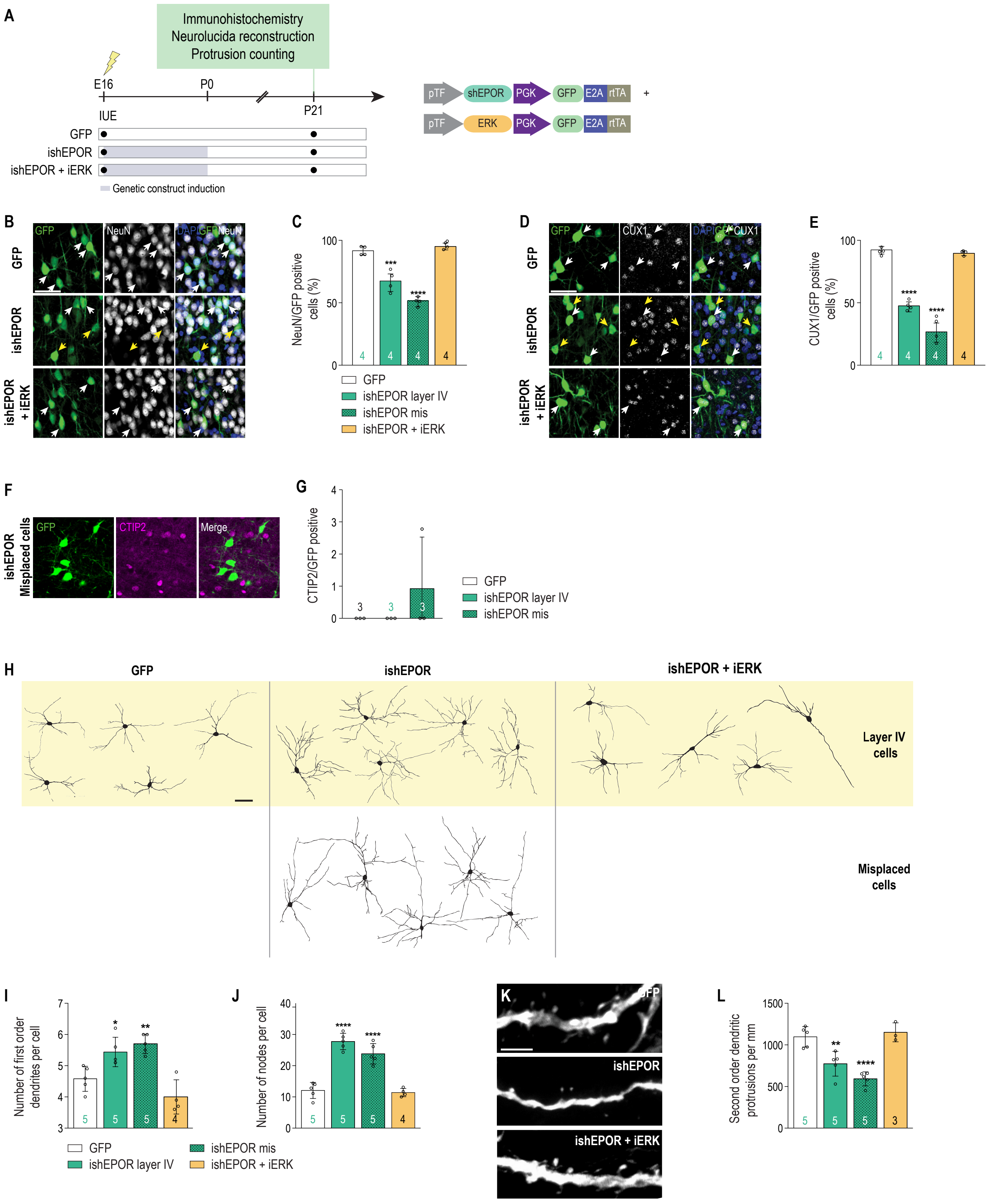

Development • Supplementary information

Figure S9. Prenatal ERK up-regulation rescues prenatal ishEPOR-induced perturbed differentiation.

(A) Experimental time-line. **(B)** NeuN-staining at P21 of control (upper panel), ishEPOR- (middle panel) and ishEPOR+iERK- (lower panel) electroporated cells. White arrows show NeuN-positive cells in all conditions and yellow arrows show NeuN-negative cells in ishEPOR-electroporated brains. **(C)** Percentage at P21 of electroporated cells positive for NeuN staining in Layer IV control, Layer IV and misplaced ishEPOR and Layer IV ishEPOR+iERK conditions (GFP: *n* = 4, ishEPOR: *n* = 4, ishEPOR+iERK: *n* = 4. One-way ANOVA followed by Bonferroni's multiple comparisons test: $F_{(3, 12)}$ = 56.88, $p < 0.0001$). **(D)** Cux1-staining at P21 of control (upper panel), ishEPOR- (middle panel) and ishEPOR+iERK- (lower panel) electroporated cells. White arrows show Cux1-positive cells in all conditions and yellow arrows show Cux1-negative cells in ishEPOR-electroporated brains. **(E)** Percentage at P21 of electroporated cells positive for Cux1 staining in Layer IV control, Layer IV and misplaced ishEPOR and Layer IV ishEPOR+iERK conditions (GFP: *n* = 4, ishEPOR: *n* = 4, ishEPOR +iERK: *n* = 4. One-way ANOVA followed by Bonferroni's multiple comparisons test: $F_{(3, 12)}$ = 104.4, $p < 0.0001$). **(F)** CTIP2-staining at P21 of misplaced ishEPOR-electroporated cells. **(G)** Percentage at P21 of electroporated cells positive for CTIP2 staining in Layer IV control, Layer IV and misplaced ishEPOR conditions (GFP: *n* = 3, ishEPOR: *n* = 3. One-way ANOVA followed by Bonferroni's multiple comparisons test: $F_{(2, 6)}$ = 0.9984, p = 0.4224). **(H)** Neurolucida morphological reconstruction at P21 of Layer IV control, Layer IV and misplaced ishEPOR and Layer IV ishEPOR+iERK conditions. **(I,J)** Quantitative morphometric analysis at P21 of Layer IV and misplaced cells at P21. (I): number of first-order dendrites per cell and (J): number of branch nodes per cell (control: *n* = 5, ishEPOR: *n* = 5, iEPORsh+iERK: *n* = 4. One-way ANOVA followed by Bonferroni's multiple comparisons test: $F_{(3, 15)}$ = 14.70 and $p <$ 0.0001 for D; $F_{(3, 15)}$ = 47.51 and $p < 0.0002$ for E). **(K)** Dendritic protrusions at P21 in Layer IV in control (upper), ishEPOR (middle) and ishEPOR+iERK (lower) conditions. **(L)** Quantification of dendritic protrusions in Layer IV control at P21, Layer IV and misplaced ishEPOR and Layer IV ishEPOR+iERK (control: *n* = 5, ishEPOR: *n* = 5, ishEPOR+iERK: *n* = 3. One-way ANOVA followed by Bonferroni's multiple comparisons test: $F_{(3, 14)}$ = 21.23, $p <$ 0.0001). Scale bars : 2 µm (K) and 50 µm (H). Error bars, SD. Abbreviations: IUE = *In utero* electroporation, *n* = number of analyzed brains (for each brain, more than 100 neurons (C and E) were considered to define the mean), ****p*<.001, *****p*<.0001.

Development • Supplementary information

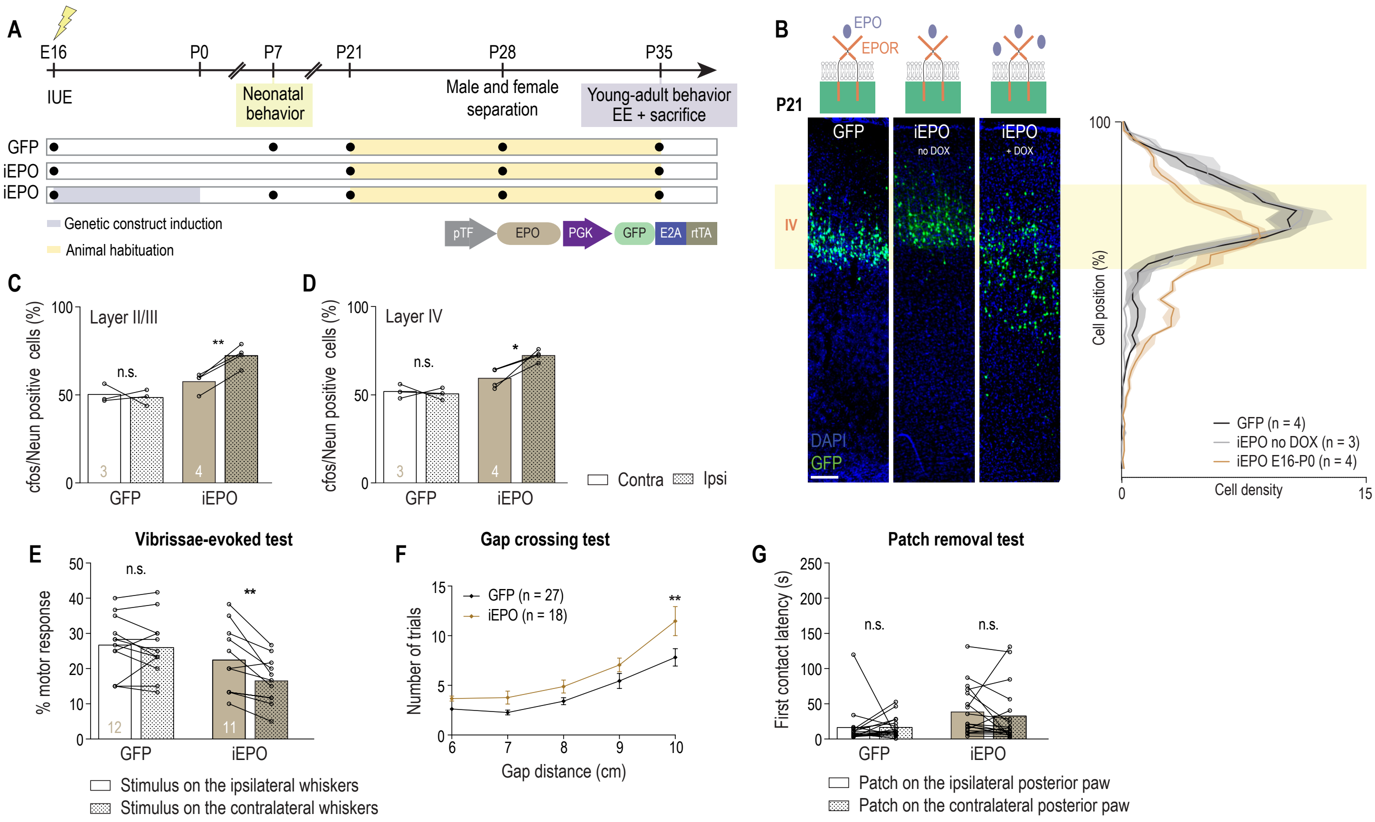

Figure S10: Pre-natal overexpression of EPO impairs neuronal positioning, neuronal activity and sensory functions later in life.

(A) Experimental time-line for cell positioning experiments. **(B)** Left panel: Coronal slices from P21 E16-electroporated brains with GFP or iEPO (non-induced and induced between E16 and P0). Right panel: Quantification of the distribution of electroporated cells along the cortex at P21 (control: *n* = 4, iEPO no DOX: *n* = 3, iEPO + DOX: *n* = 4. Two-way ANOVA followed by Bonferroni's multiple comparisons test: $F_{(78, 312)} = 3.747$, $p < 0.0001$; cell position main effect: $F_{(39, 312)} = 65.2$, $p < 0.0001$; group main effect: $F_{(2, 8)} = 1.827$, $p = 0.2220$). **(C)** Percentage of cFos-positive NeuN cells in Layer II-III in control and iEPO conditions after enriched environment at P35 (GFP: *n* = 3 brains, iEPO: *n* = 4. Paired t-test: $p = 0.9665$ for GFP, $p = 0.0023$ for iEPO). **(D)** Percentage of cFos-positive NeuN cells in Layer IV in control and iEPO conditions after enriched environment at P35 (GFP: *n* = 3 brains, iEPO: *n* = 4. Paired t-test: $p = 0.6885$ for GFP, $p = 0.0287$ for iEPO). **(E)** Percentage of motor response during vibrissae-evoked behavior test at P7 for both sides for control and iEPO conditions (GFP: 12 animals, iEPO: 11 animals. Paired t-test: $p = 0.5961$ for GFP; $p = 0.0041$ for iEPO). **(F)** Number of trials to cross the gap in function of the distance of the gap for GFP and iEPO groups at P35 (GFP: 27 animals, iEPO: 18 animals. Two-way ANOVA followed by Bonferroni's multiple comparisons test: gap x group interaction: $F_{(4, 154)} = 1.064$, $p = 0.3765$; gap main effect: $F_{(4, 154)} = 32{,}41$, $p < 0.0001$; group main effect: $F_{(1, 154)} = 19.49$, $p < 0.0001$). **(G)** First contact latency in patch-removal task at P35 for both sides for control and iEPO conditions (GFP: 27 animals, iEPO: 18 animals. Paired t-test: $p = 0.9940$ for GFP; $p = 0.4471$ for iEPO). The GFP conditions shown in B, in C-D and in E-G are reported from Fig. 2D, Fig. 6 and Fig. 7, respectively. Scale bar : 100 µm (B). Error bars, SEM. Abbreviations: IUE = *In utero* electroporation, *n* = number of analyzed brains (for each brain more than 200 neurons (B) were considered to define the mean) or number of mice (E-G), $^{**}p<.01$, $^{***}p<.001$, $^{****}p<.0001$.

Table S1. Expression plasmids used in this study

Abbreviation	Full name	Components	Full sequence and map	Parental plasmid
UBI-GFP	pCLX-UBI-GFP	UBI promoter, GFP gene	http://lentilab.unige.ch/plasmidbase.html	
PG-EPOR	pCWXPG-UBI-ratEPOR	PGK promoter, GFP gene, UBI promoter, rat EPO receptor gene	http://lentilab.unige.ch/plasmidbase.html	GE-Healthcare-Bio-Sciences AB Cat. MRN1768 29123514
PG-shEPOR	pCWXPG-UBI-mirGE-ratEPOR3x	PGK promoter, GFP gene, UBI promoter, anti-rat EPO receptor mirRNA	http://lentilab.unige.ch/plasmidbase.html	
PGR-shEPOR	pCWXPGR-pTF-mirGE-ratEPOR3x	Auto-inducible with GFP gene, anti-rat EPO receptor mirRNA	http://lentilab.unige.ch/plasmidbase.html	
EBR-shEPOR	pCLX-pTF-mirGE-ratEPOR3x-EBR	Auto-inducible with BSD gene, anti-rat EPO receptor mirRNA	http://lentilab.unige.ch/plasmidbase.html	
PG-EPO	pCWXPG-UBI-ratEPO	PGK promoter, GFP gene, UBI promoter, rat EPO gene	http://lentilab.unige.ch/plasmidbase.html	pCAGS-ratEpo https://www.ncbi.nlm.nih.gov/pubmed/10697117
PGR-EPO	pCWXPGR-pTF-ratEPO	Auto-inducible with GFP gene, rat EPO gene	http://lentilab.unige.ch/plasmidbase.html	
PG-shEPO	pCWXPG-UBI-mirGE-ratEpo3x	PGK promoter, GFP gene, UBI promoter, anti-rat EPO mirRNA	http://lentilab.unige.ch/plasmidbase.html	
PGR-shEPO	pCWXPGR_pTF-mirGE-ratEpo3x	Auto-inducible with GFP gene, anti-rat EPO mirRNA	http://lentilab.unige.ch/plasmidbase.html	
PC-ERK	pCWXPC-UBI-ERKMEK	PGK promoter, mCherry gene, UBI promoter, ERK::MEK fusion protein	http://lentilab.unige.ch/plasmidbase.html	pCMV-myc-ERK2-L4A-MEK1_fusion **addgene #39197**
PGR-ERK	pCWXPGR-pTF-ERKMEK	Auto-inducible with GFP gene,	http://lentilab.unige.ch/plasmidbase.html	

Development • Supplementary information

Abbreviation	Full name	Components	Full sequence and map	Parental plasmid
		ERK::MEK fusion protein		
PC-Akt	pCWXPC-UBI-AKT	PGK promoter, mCherry gene, UBI promoter, Akt protein	http://lentilab.unige.ch/plasmidbase.html	pcDNA3 Myr HA Akt1 Addgene #9008
PGR-Akt	pCWXPGR-pTF-AKT	Auto-inducible with GFP gene, Akt protein	http://lentilab.unige.ch/plasmidbase.html	
EKAR	nuclear EKAR (EGFP-mRFP)	ERK activity reporter	Addgene #18682	

Table S2. Oligos used for the cloning of anti-EPO and anti-EPOR mirGE

mirGE microRNA against rat EPO	ggatccatcgatactagtGGTGATAGCAATGTCAGCAGTGCCT**GAAAGTATCCGCTGTGAGTGTT**GTGAAGCCACAGATG**AACACTCACAACGGATACTTTA**AAGTAAGGTTGACCATACTCTACtctaga
mirGE microRNA against rat EPOR	ggatccatcgatactagtGGTGATAGCAATGTCAGCAGTGCCT**ATGTTTCTGAACCTTCATCCAT**GTGAAGCCACAGATG**ATGGATGAAGATTCAGAAACAC**AAGTAAGGTTGACCATACTCTACtctaga

Targeting (antisense) strands are in uppercase bold underlined, guide strands are in uppercase bold, stem and loop strands are in uppercase, and sequences for cloning containing BamHI, SpeI and XbaI sites are in lowercase. Details on mirGE design and cloning can be found in (Giry-Laterrière et al., 2011).

Development • Supplementary information

Table S3. Oligos used for RNA quantification

Rat EPO qPCR sense	AAGTTTGGCAAGGCCTGTCT
Rat EPO qPCR antisense	TATCCGCTGTGAGTGTTCGG
	Amplicon size 224 bp
Rat EPO-R qPCR sense	CGTCGAGTTTTGTGCCACTG
Rat EPO-R qPCR antisense	GGTTGCTCAGGACACACTCA
	Amplicon size 287 bp
Rat riboprobe EPO sense SP6	cgatgtatttaggtgacactatagaaAAGTTTGGCAAGGCCTGTCT
Rat riboprobe EPO antisense T7	cgatgttaatacgactcactatagggTATCCGCTGTGAGTGTTCGG
	Amplicon size 225 bp
Rat riboprobe EPO-R sense SP6	cgatgtatttaggtgacactatagaaGAAAGTCATGTCGCCTGCAC
Rat riboprobe EPO-R antisense T7	cgatgttaatacgactcactatagggGACCTCCACCCTTTGTGTCC
	Amplicon size 332 bp
Human cyclophilin A sense	CCATTTGTGTTGGGTCCAGC
Human cyclophilin A antisense	TACGGGTCCTGGCATCTTGT
	Amplicon size 50 bp
Rat cyclophilin A sense	AAATGCCCGCAAGTCAAAGA
Rat cyclophilin A antisense	TCACCATCTCCGACTGTGGA
	Amplicon size 50 bp

Sequence-specific nucleotides are in uppercase.

Development • Supplementary information
