# Supplementary material for: Endogenous erythropoietin signaling regulates migration and laminar positioning of upper-layer neurons in the developing neocortex
Source: Development. 2020 Oct 7;147(19):dev190249. doi: 10.1242/dev.190249 (PMC7561482; doi:10.1242/dev.190249)
Supplement: Reviewer comments [file develop-147-190249_review_history.pdf]

Endogenous erythropoietin signaling regulates migration and laminar positioning of upper-layer neurons in the developing neocortex

Paul E. Constanthin, Alessandro Contestabile, Volodymyr Petrenko, Charles Quairiaux, Patrick Salmon, Petra S. Hüppi and Jozsef Zoltan Kiss
DOI: 10.1242/dev.190249

Editor: Paola Arlotta

Review timeline
Original submission: 5 March 2020
Editorial decision: 14 April 2020
First revision received: 18 June 2020
Accepted: 24 July 2020

Original submission

First decision letter

MS ID#: DEVELOP/2020/190249

MS TITLE: Endogenous erythropoietin signaling regulates migration and laminar positioning of upper-layer neurons in the developing neocortex

AUTHORS: Paul E. Constanthin, Alessandro Contestabile, Volodymyr Petrenko, Charles Quairiaux, Patrick Salmon, Petra S. Hüppi, and Jozsef Zoltan Kiss

I have now received all the referees' reports on the above manuscript, and have reached a decision. The referees' comments are appended below, or you can access them online: please go to BenchPress and click on the 'Manuscripts with Decisions' queue in the Author Area.

As you will see, the referees express considerable interest in your work, but have some significant criticisms and recommend a substantial revision of your manuscript before we can consider publication. If you are able to revise the manuscript along the lines suggested, which may involve further experiments, I will be happy receive a revised version of the manuscript. Your revised paper will be re-reviewed by one or more of the original referees, and acceptance of your manuscript will depend on your addressing satisfactorily the reviewers' major concerns. Please also note that Development will normally permit only one round of major revision.

We are aware that you may currently be unable to access the lab to undertake experimental revisions. If it would be helpful, we encourage you to contact us to discuss your revision in greater detail. Please send us a point-by-point response indicating where you are able to address concerns raised (either experimentally or by changes to the text) and where you will not be able to do so within the normal timeframe of a revision. We will then provide further guidance. Please also note that we are happy to extend revision timeframes as necessary.

Please attend to all of the reviewers' comments and ensure that you clearly highlight all changes made in the revised manuscript. Please avoid using 'Tracked changes' in Word files as these are lost in PDF conversion. I should be grateful if you would also provide a point-by-point response detailing how you have dealt with the points raised by the reviewers in the 'Response to Reviewers' box. If you do not agree with any of their criticisms or suggestions please explain clearly why this is so.

© 2020. Published by The Company of Biologists under the terms of the Creative Commons Attribution License (https://creativecommons.org/licenses/by/4.0/).

Reviewer 1

Advance summary and potential significance to field

Review on “Endogenous erythropoietin signaling regulates migration and laminar positioning of upper-layer neurons in the developing neocortex” by Constanthin, Contestabile et al.
This article describes a novel molecular mechanism involved in the proper generation of the neocortex:
EPO/EPOR interaction contributes to the migration and positioning of superficial cortical neurons (layers II to IV). Interestingly, this molecular interaction mediates two crucial cellular steps in neuronal migration, 1) the multipolar-to-bipolar transition that helps neurons to leave the intermediate zone, and 2) the active glial-
guided locomotion. Moreover, the authors discover that EPO molecular signaling requires ERK as the downstream effector target. Importantly, the potential treatment uses of EPO are presented and discussed.
The research presented covers an experimental tour-de-force, which in-depth investigates neuronal radial migration: from the molecular mechanism to the cellular behavior, and from this to the anatomical and behavioral consequences. It is impeccable at the methodological level, all tools are well designed and thought through; the battery of genetic tools well serves for the purpose of the research.
This manuscript is carefully written, all figures are admirably presented, and the methods are described in full detail. I can only acknowledge the authors for delivering such a polished work.

Comments for the author

Despite some minor weak points and concerns, which of course every manuscript presents, I consider the article a relevant piece of research, its conclusions robust and important. The following are merely a list of questions and concerns that came during my revision:

The article connects EPO/EPOR mechanism to granular and supragranular neuron development only, but I am afraid that nothing is done to exclude its action on infragranular development. The ISH data presented for both EPO and EPOR (Fig. 1) seems insufficient to exclude this action. Infragranular layers are generated prior to E16 in rat when the first ISHs are presented. I would suggest testing equivalent shEPOR electroporations on an earlier stage (E14?). If deep layer generated neurons are then misplaced as well EPO/EPOR would turn a global pan-cortical contributor to radial migration. But if properly located, the specificity of this molecular mechanism on upper layer development would be evidenced.

In several parts of the manuscript, the authors interpret the effect of EPO/EPOR misfunction as a delay of positioning. I am not entirely convinced of this. As far as I understand, if migrating neurons get delayed these would not reach their layer target, but the layer target of the neurons generated afterward. So, if neurons get delayed, should not they reach the most superficial layers (II and III), instead of the deeper ones?
In my opinion, EPO/EPOR LOF does not cause delay, but a diminished interrupted or aberrant migration.

The identity of the aberrantly positioned neurons could give interesting hints on the actual effect of EPO/EPOR signaling on brain development. There are several options regarding what those neurons may become. Those neurons which end up located at infragranular layers, do they become infragranular neurons?
In this case, EPO/EPOR would be involved in the migration mechanism only, leaving the cell identity differentiation role to the environmental cues present at deep layers. But those neurons may still become layer IV neurons that happened to be misplaced only. Or they could be delayed into a later phenotype (LII/III).
Finally, EPO/EPOR signaling could be involved in neuronal differentiation itself, and its failure may lead neurons to become something different to the expected layer-specific neurons. I would suggest to include a short study on the neuronal phenotype of those mislocated neurons after shEPOR. A few cortical layer markers could be used, such as Brn2, CTIP2 or Otx1. Specifically, RorB

© 2020. Published by The Company of Biologists under the terms of the Creative Commons Attribution License (https://creativecommons.org/licenses/by/4.0/).

should be investigated as highly specific of layer IV neurons. If possible, and given shEPOR electroporations also carry EGFP expression, I would suggest to include a brief description of the efferent projections of the misplaced neurons.

Indeed, RorB is the key marker. The authors continuously refer to layer IV neurons but have not tested layer IV identity in the whole article (marker expression, efference pattern, neuronal morphology) besides location in the cortex evaluated with DAPI.

Regarding cortical layer markers, Satb2 is mentioned and used in the article as a marker of SVZ progenitors (intermediate precursors; p7, line 7). Satb2 does not label intermediate precursor cells, but postmitotic neurons (occupying all cortical layers or only upper ones, depending on cortical area). This should be considered in the description of the results.

It makes no much of a sense to me that EPO levels must be adequate for proper cortical development
(mentioned in several places, especially p13, lines 24-25; p15, lines 16-18), but its downstream effector ERK activity can over-function (as in the ERK overexpression experiments) and the cortex develops normally. Is it not a contradiction? Please discuss the potential reasons behind this inconsistency.

The use of the terms “dorsal” and “ventral” is wrong in the anatomical context (p8, line 21). The right sentence should be “We also observed a slightly higher proportion of ishEPOR electroporated cells even presenting an inversion of the leading process from the basal (i.e. towards pial surface) to apical (i.e. towards ventricle) direction,…”. For reference, please check this terminology on Boulder Committee, 1970.

Figure 1 would benefit of layer annotation on the two P7 images.

I miss a couple of standard controls that, if have been performed already, should be presented. When short hairpins are used (widely and wisely throughout the article), shControl experiments are usually required to exclude any off-target effect, most common ones are scrambled and non-targeting sh controls. In addition in the conditional experiments, it is not clear to me whether GFP-only control experiments were conducted with the comparable DOX control administration, to test any DOX effect out.

Figure 6B and 7C are identical. Please check whether it should be the case.

There is no figure S10, I guess it is the one named 11.

<u>Reviewer 2</u>

Advance summary and potential significance to field

The paper examines the role that EPO receptors play in the migration and positioning of cortical neurons in layer IV of S1 and then examine the impact on sensory-related behaviors. The approaches revolved principally around the use of IUE to introduce constitutively expressed or Dox-controlled expression of a shRNA to knockdown receptor or ligand levels. They include a variety of measures to evaluate the outcomes including the evaluation and manipulation of ERK activity--a candidate downstream signaling pathway. The hypothesis is underexplored, the findings are intriguing and it was genuinely unexpected that long term CA-ERK overexpression appeared to rescue the migratory phenotype observed with EPOR knockdown. Concerns about the reagents used undermine what would be a solid addition to the field.

Comments for the author

The key concern is whether or not the main reagents (miRNA-based, exogenously introduced shRNAs) are selectively striking their targets. Some controls are presented in supplementary data, but they are incomplete and given their importance, should be shown within the main figures. Outside of this major issue, other concerns are minor.

© 2020. Published by The Company of Biologists under the terms of the Creative Commons Attribution License (https://creativecommons.org/licenses/by/4.0/).

1. One shRNA per target is identified and tested by knockdown of overexpressed protein in HEK cells. Given that off target effects with such reagents is common, additional control reagents are required in order to be confident that the data reflect decreased receptor/ligand. This could be done by inclusion of an additional shRNA targeting a different sequence on the same receptor (and showing similar outcomes) or by coupling knockdown with overexpression of the receptor in the same cells or by inclusion of an alternate strategy. Not every datapoint needs to be repeated, but more is needed. GFP alone is not a sufficient control as it does not activate interferon and other pathways known to be induced by shRNAs. This issue is also not fully addressed by the Dox regulated construct because when the shRNA is not expressed it is also not producing potential off-target effects.

2. The controls for the shRNAs that are provided in supplementary data are also puzzling. In supp figure 1C it appears that EPOR mRNA is broadly diminished throughout the left hemisphere in a manner that is much broader than might be expected and not fully consistent with the effects of targeted knockdown shown in the other figures. In supp figure 1E, the immunolabeling interpretation is also not clear. If layer IV cells are misplaced in layer V following knockdown, why do they still express EPOR at the same level in cells with GFP as those without GFP. Is the antibody specific? Similarly, EPO overexpression is verified in tissue (supp 7D)
but not its knockdown.

3. There are no data that show time course of DOX activation. This is of secondary importance compared to the first two items, but would be useful in order to fully interpret the data as the impact is more modest than the constitutive version and could be due to the restricted time period or to the promoter.

4. Differences used to distinguish between multipolar and bipolar cells are not evident in the figures (e.g. 3B and 4D).

5. EPO overexpression/knockdown data do not support autocrine action and it would be helpful to provide a working model/s incorporating sites of ligand/receptor interaction.

6. While cortical migration is normally complete at P21, the data presented in figure 2 suggest that EPOR knockdown cells may complete their migration at a later stage. "Permanent" (summary and discussion) goes beyond the data.

First revision

Author response to reviewers' comments

Reviewer #1

1) “Despite some minor weak points and concerns, which of course every manuscript presents, I consider the article a relevant piece of research, its conclusions robust and important.”

We thank the Reviewer for the positive note.

2) “The article connects EPO/EPOR mechanism to granular and supragranular neuron development only, but I am afraid that nothing is done to exclude its action on infragranular development. The ISH data presented for both EPO and EPOR (Fig. 1) seems insufficient to exclude this action. Infragranular layers are generated prior to E16 in rat when the first ISHs are presented.”

Indeed, in this study we focused on the migration of granular and supragranular neurons and in the absence of relevant data, we did not intent to exclude the role of EPO signaling in lower layer pyramidal neuron migration. Accordingly, this fact is clearly stated in the title of our manuscript. Nevertheless, we agree that the role of EPO signaling in lower layers also needs to be investigated.

© 2020. Published by The Company of Biologists under the terms of the Creative Commons Attribution License (https://creativecommons.org/licenses/by/4.0/).

Noteworthy that ERK signaling has been suggested to play a different role in lower and upper layers (Xing et al. 2016). It is thus possible that this signaling might be involved in different functions in lower layer neurons that needs to be addressed in detail by future studies.

3) "In several parts of the manuscript, the authors interpret the effect of EPO/EPOR misfunction as a delay of positioning. I am not entirely convinced of this. As far as I understand, if migrating neurons get delayed, these would not reach their layer target, but the layer target of the neurons generated afterward. So, if neurons get delayed, should not they reach the most superficial layers (II and III), instead of the deeper ones? In my opinion, EPO/EPOR LOF does not cause delay, but a diminished interrupted or aberrant migration."

In our view, the migration of neurons could be either be permanently disrupted/arrested or just delayed (in timing), in which case neurons may eventually reach their final destination. We observed this later phenomenon in the developing cortex after transient decrease of the canonical Wnt signaling (Bocchi et al., 2017). We agree with the Referee, that our data clearly show a permanent mispositioning of a population of neurons, hence the migration is interrupted before neurons reached layer IV. In view of the different degree of the efficiency of loss-of-function in individual neurons, we cannot exclude the possibility that the migration of some neurons could be just delayed. Nonetheless, to avoid misinterpretation of these results and to accommodate the suggestion of the Referee, we have changed the term migration delay into interrupted or aberrant migration throughout the manuscript.

4) "The identity of the aberrantly positioned neurons could give interesting hints on the actual effect of EPO/EPOR signaling on brain development. There are several options regarding what those neurons may become. Those neurons which end up located at infragranular layers, do they become infragranular neurons? In this case, EPO/EPOR would be involved in the migration mechanism only, leaving the cell identity differentiation role to the environmental cues present at deep layers. But those neurons may still become layer IV neurons that happened to be misplaced only. Or they could be delayed into a later phenotype (LII/III). Finally, EPO/EPOR signaling could be involved in neuronal differentiation itself, and its failure may lead neurons to become something different to the expected layer-specific neurons. I would suggest to include a short study on the neuronal phenotype of those mislocated neurons after shEPOR. A few cortical layer markers could be used, such as Brn2, CTIP2 or Otx1. Specifically, RorB should be investigated as highly specific of layer IV neurons. If possible, and given shEPOR electroporations also carry EGFP expression, I would suggest to include a brief description of the efferent projections of the misplaced neurons."

We fully agree with this opinion. Our data show that neurons of the future layer IV electroporated with shEPOR do not display an altered early neuronal differentiation (SATB2 and TBR2 percentage of neurons are similar to the control group). On the other hand, we observed that an important percentage of EPOR loss-of-function neurons in layer IV, and also misplaced neurons lower layers, are not stained CUX1 and NeuN. Therefore, we think that EPO signaling is involved in neuronal migration as well as in the last steps of neuronal differentiation. In order to address the point raised by the Referee, we performed CTIP2 staining on P21 slides obtained from E16 electroporated brains. We found no difference in the percentage of electroporated cells positive to CTIP2 between GFP control, shEPOR layer IV and shEPOR misplaced group (see in new sup Fig. 9). These results suggest that the position of misplaced neurons in lower layers does not affect the neuronal phenotype. We discuss this point in a new paragraph in the revised Discussion page 16, line 23-27.

Moreover, as suggested by the Reviewer, we added two new sets of data obtained by P21 neuronal reconstruction and protrusion density analysis performed on layer IV and misplaced electroporated neurons (see new sup. figure 9 and new text in Results, page 11-12). We found an aberrant morphology and a decreased protrusion density in ishEPOR neurons. Importantly, ERK overexpression fully rescued these alterations. Together, these results give support to the hypothesis that transient down-regulation of EPO signaling affects late steps of neuronal differentiation including dendritic development and the formation of synapses.

5) "Indeed, RorB is the key marker. The authors continuously refer to layer IV neurons but have not tested layer IV identity in the whole article (marker expression, efference pattern, neuronal morphology) besides location in the cortex evaluated with DAPI."

© 2020. Published by The Company of Biologists under the terms of the Creative Commons Attribution License (https://creativecommons.org/licenses/by/4.0/).

We have carried out several immunostaining experiments using a RorB antibody, but unfortunately, we repeated failed to obtain satisfying signal in layer IV. However, in a previous publication from our lab, we have demonstrated that our protocol of electroporation (electroporation at E16) is specifically targeting layer IV. We include this reference in the revised manuscript (Petrenko et al. 2015).

6) "Regarding cortical layer markers, Satb2 is mentioned and used in the article as a marker of SVZ progenitors (intermediate precursors; p7, line 7). Satb2 does not label intermediate precursor cells, but postmitotic neurons (occupying all cortical layers or only upper ones, depending on cortical area). This should be considered in the description of the results."

We thank the Reviewer for this clarification. We have modified the description of the results accordingly (pages 6 and 16).

7) "It makes no much of a sense to me that EPO levels must be adequate for proper cortical development (mentioned in several places, especially p13, lines 24-25; p15, lines 16-18), but its downstream effector ERK activity can over-function (as in the ERK overexpression experiments) and the cortex develops normally. Is it not a contradiction? Please discuss the potential reasons behind this inconsistency."

Our results indicate that in fact after over-function of ERK the cortex does not develop normally. This manipulation leads to an over-migration of the neurons that will start to populate layer II-III (see Sup. Fig. 8C-D and manuscript page 9, line 30-32). In order to better explain this point, we have modified the text in page 9.

8) "The use of the terms "dorsal" and "ventral" is wrong in the anatomical context (p8, line 21). The right sentence should be "We also observed a slightly higher proportion of ishEPOR electroporated cells even presenting an inversion of the leading process from the basal (i.e. towards pial surface) to apical (i.e. towards ventricle) direction,…". For reference, please check this terminology on Boulder Committee, 1970."

We thank Reviewer 1 for this clarification. We have corrected the text accordingly.

9) "Figure 1 would benefit of layer annotation on the two P7 images."

We thank the Reviewer for this suggestion; we have modified the figure as suggested.

10) "I miss a couple of standard controls that, if have been performed already, should be presented. When short hairpins are used (widely and wisely throughout the article), shControl experiments are usually required to exclude any off-target effect, most common ones are scrambled and non-targeting sh controls. In addition, in the conditional experiments, it is not clear to me whether GFP-only control experiments were conducted with the comparable DOX control administration, to test any DOX effect out."

We thank the Reviewer for this pertinent remark. In a previous experimental series in the lab, we have performed such control experiments. We have produced shRNAs to mRNA sequences unrelated to EPOR, such as the chemokine receptor 5 (CCR5) that has no known function in neuronal migration. We electroporated control plasmids at E18 targeting upper layer neurons and the effects on cell positioning has been analyzed. The results did not reveal any significant differences in cell positioning between this off-target shRNA and the GFP-overexpressing plasmid used as control in this study (new Sup. Fig 1C, page 5 and 22). While, electroporation at the same time point performed with an shEPOR clearly showed a perturbation of cell positioning (Sup. Fig. 3).

GFP-only control experiments were conducted with no DOX administration as reported in the manuscript. We previously reported that DOX per se do not have any effect (Giry-Laterrière et al., 2011).

11) "Figure 6B and 7C are identical. Please check whether it should be the case."

© 2020. Published by The Company of Biologists under the terms of the Creative Commons Attribution License (https://creativecommons.org/licenses/by/4.0/).

We thank the Reviewer for this note. These two figures are not identical. The control group is the same, but not the experimental group.

12) “There is no figure S10, I guess it is the one named 11.”

We are sorry for this error, we corrected it accordingly.

Reviewer #2

1) “One shRNA per target is identified and tested by knockdown of overexpressed protein in HEK cells. Given that off target effects with such reagents is common, additional control reagents are required in order to be confident that the data reflect decreased receptor/ligand. This could be done by inclusion of an additional shRNA targeting a different sequence on the same receptor (and showing similar outcomes) or by coupling knockdown with overexpression of the receptor in the same cells or by inclusion of an alternate strategy. Not every data point needs to be repeated, but more is needed. GFP alone is not a sufficient control as it does not activate interferon and other pathways known to be induced by shRNAs. This issue is also not fully addressed by the Dox regulated construct because when the shRNA is not expressed it is also not producing potential off-target effects. “

We thank Reviewer 2 for these pertinent suggestions. We did not specify in the original manuscript that both EPOR and EPO shRNA plasmid were ordered from Santa Cruz Biotechnology. The original products were a pool of 2-3 target-specific lentiviral vector plasmids each encoding 19-25 nucleotides (plus hairpin) designed to knock down gene expression. In order to minimize the volume of the original pool of plasmids, the plasmids were separated and their efficacy was tested in vivo through in utero electroporation. The plasmids showed a significant effect on cellular position, some more than others. After selection of the most efficient shEPO and shEPOR plasmid, they were then modified as explained in materials and methods chapter. This clarification was added to the Materials and Methods and the proportion of electroporated cells that reached layer IV after being electroporated with the different plasmids was indicated (page 18).

Concerning the controls for off-target effects of plasmids see answer to Reviewer 1 (question number 9), where we describe the experiment with off-target shRNA. The date from this control experiment have been added to Sup. Fig 1C.

2) “The controls for the shRNAs that are provided in supplementary data are also puzzling. In supp figure 1C, it appears that EPOR mRNA is broadly diminished throughout the left hemisphere in a manner that is much broader than might be expected and not fully consistent with the effects of targeted knockdown shown in the other figures. In supp figure 1E, the immunolabeling interpretation is also not clear. If layer IV cells are misplaced in layer V following knockdown, why do they still express EPOR at the same level in cells with GFP as those without GFP. Is the antibody specific?”

We thank the reviewer for this remark. Concerning Sup. figure 1C (Sup. Fig 1 D in the revised version), it is possible to have such a broader electroporation involving a large population of the future somatosensory cortex. Unfortunately, we were not able to verify the spread of the electroporation because of the ISH protocol. It should be mentioned that lower layers are more affected by the shEPOR than upper layers where the majority of migrating cells not yet arrived at E19.

Concerning Sup. Figure 1E, this figure shows immunostaining with specific EPOR antibody of GFP- (upper panel) and shEPOR- (lower panel) electroporated neurons located in layer IV. One can observe two shEPOR-electroporated neurons, one EPOR negative and the second EPOR positive. It should be emphasized that here we performed a qualitative and not a quantitative analysis of EPOR immunostaining.

3) “There are no data that show time course of DOX activation. This is of secondary importance compared to the first two items, but would be useful in order to fully interpret the data as the

© 2020. Published by The Company of Biologists under the terms of the Creative Commons Attribution License (https://creativecommons.org/licenses/by/4.0/).

impact is more modest than the constitutive version and could be due to the restricted time period or to the promoter.”

We added in the manuscript (pages 6 and 19) a reference of a previous published paper from the lab reporting the time course of DOX activation.

4) “Differences used to distinguish between multipolar and bipolar cells are not evident in the figures (e.g. 3B and 3I).”

We thank the Reviewer for this remark. To improve the quality of illustration in Fig 3B and 3I we added new photomicrographs.

5) “EPO overexpression/knockdown data do not support autocrine action and it would be helpful to provide a working model/s incorporating sites of ligand/receptor interaction.”

Without claiming that EPO ligand have a complete autocrine action, we speculate that such an action is possible and we also discussed a possible paracrine function of near neurons (see Discussion, pages 14-15).

6) While cortical migration is normally complete at P21, the data presented in figure 2 suggest that EPOR knockdown cells may complete their migration at a later stage. "Permanent" (summary and discussion) goes beyond the data.

The results presented in Figure 2 reveal that about 60% of shEPOR-electroporated neurons fail to reach layer IV at P0 as well as P21 indicating a permanent mispositioning. This conclusion is further confirmed by data from cell positioning analysis at P35 that are comparable to that observed at P21 in figure 2 (data not shown). We have modified the text accordingly (Results, page 6, line 10-11)

Second decision letter

MS ID#: DEVELOP/2020/190249

MS TITLE: Endogenous erythropoietin signaling regulates migration and laminar positioning of upper-layer neurons in the developing neocortex

AUTHORS: Paul E. Constanthin, Alessandro Contestabile, Volodymyr Petrenko, Charles Quairiaux, Patrick Salmon, Petra S. HÃ¼ppi, and Jozsef Zoltan Kiss
ARTICLE TYPE: Research Article

I am happy to tell you that your manuscript has been accepted for publication in Development, pending our standard ethics checks.

Reviewer 1

Advance summary and potential significance to field

This article describes a novel molecular mechanism involved in the proper generation of the neocortex: EPO/EPOR interaction contributes to the migration and positioning of superficial cortical neurons (layers II to IV). Interestingly, this molecular interaction mediates two crucial cellular steps in neuronal migration, 1) the multipolar-to-bipolar transition that helps neurons to leave the intermediate zone, and 2) the active glial-guided locomotion. Moreover, the authors discover that EPO molecular signaling requires ERK as the downstream effector target. Importantly, the potential treatment uses of EPO are presented and discussed.

© 2020. Published by The Company of Biologists under the terms of the Creative Commons Attribution License (https://creativecommons.org/licenses/by/4.0/).

Comments for the author

The authors have completed a thorough revision fulfilling all my queries and comments.

Reviewer 2

Advance summary and potential significance to field

The findings will be of broad interest to the field.
1. That EPO signaling plays a role in neural migration is novel.
2. The data underscore that even transient signaling disruptions during brain development can have lasting consequences.

Comments for the author

The authors have addressed the outstanding issues that were raised in my critique. In particular, the inclusion of additional data addressing on-target and off-target shRNA effects alleviated concerns about the main reagents used throughout. However, I agree with R1 that it is puzzling to see data repeated in different figures. While it is acknowledged in the response that the first three (control) panels in 6B are indeed the first three (control) panels in 7C, such repetition is usually the result of an error, and re-use, at a minimum should be noted directly in the figure legend to reduce confusion. Ideally, another image would be used.

© 2020. Published by The Company of Biologists under the terms of the Creative Commons Attribution License (https://creativecommons.org/licenses/by/4.0/).
